# Cost-effectiveness of angiography-guided thyroidectomy to prevent postoperative hypoparathyroidism: Spanish National Health System model-based analysis

**DOI:** 10.1093/bjsopen/zrag095

**Published:** 2026-07-22

**Authors:** Pablo Moreno-Llorente, Marta Ruiz, Arantxa García-Barrasa, Marta Recasens-Subias, Itziar Larrañaga, Thiago Carnaval, Sebastián Videla

**Affiliations:** Unidad de Cirugía Endocrina, Hospital Universitari de Bellvitge, L’Hospitalet de Llobregat, Barcelona, Spain; Departament de Ciències Clíniques, Facultat de Medicina i Ciències de la Salut, Universitat de Barcelona, Barcelona, Spain; Unidad de Cirugía Endocrina, Hospital Universitari de Bellvitge, L’Hospitalet de Llobregat, Barcelona, Spain; Unidad de Cirugía Endocrina, Hospital Universitari de Bellvitge, L’Hospitalet de Llobregat, Barcelona, Spain; Unidad de Cirugía Endocrina, Hospital Universitari de Bellvitge, L’Hospitalet de Llobregat, Barcelona, Spain; Unidad de Cirugía Endocrina, Hospital Universitari de Bellvitge, L’Hospitalet de Llobregat, Barcelona, Spain; Methodological and Statistical Support Department, Fundació de Recerca Sant Joan de Déu, Esplugues de Llobregat, Barcelona, Spain; Pharmacology Unit, Department of Pathology and Experimental Therapeutics, School of Medicine and Health Sciences, Institute of Neurosciences, University of Barcelona, L'Hospitalet de Llobregat, Spain; Neuropharmacology and Pain Group, Neuroscience Program, Bellvitge Biomedical Research Institute, L’Hospitalet de Llobregat, Barcelona, Spain; Pharmacology Unit, Department of Pathology and Experimental Therapeutics, School of Medicine and Health Sciences, Institute of Neurosciences, University of Barcelona, L'Hospitalet de Llobregat, Spain; Neuropharmacology and Pain Group, Neuroscience Program, Bellvitge Biomedical Research Institute, L’Hospitalet de Llobregat, Barcelona, Spain; Clinical Research Support Area, Germans Tries I Pujol University Hospital, Badalona, Spain

**Keywords:** endocrine surgery, indocyanine green, health technology assessment, near-infrared imaging

## Abstract

**Background:**

Postoperative hypoparathyroidism is the most common complication after total thyroidectomy, and is associated with impaired quality of life and increased long-term healthcare use. Indocyanine green fluorescence imaging enables real-time intraoperative assessment of parathyroid perfusion, potentially improving gland preservation. However, its cost-effectiveness in routine surgical practice has not been formally evaluated.

**Methods:**

A model-based cost-effectiveness analysis was conducted comparing angiography-guided thyroidectomy with conventional thyroidectomy from the perspective of the Spanish National Health System. A state-transition Markov model simulated long-term outcomes for adults undergoing thyroidectomy, incorporating the incidence of transient and permanent hypoparathyroidism, health-related quality-adjusted life-years, and direct medical costs. The model used contemporary surgical data, included a 35-year time horizon, and discounted costs and effects at 3.5% annually. Probabilistic and deterministic sensitivity analyses were used to assess parameter and structural uncertainty. Cost-effectiveness was judged at a willingness-to-pay threshold of €30 000 per quality-adjusted life-year.

**Results:**

Angiography-guided thyroidectomy was associated with lower lifetime healthcare costs (€563.7 million *versus* €613.2 million) and greater health benefits (310 997 *versus* 299 107 quality-adjusted life-years), yielding a gain of 11 889 quality-adjusted life-years and €49.5 million in cost savings. Angiography-guided thyroidectomy dominated conventional thyroidectomy in all sensitivity analyses, with an incremental net monetary benefit of €406.2 million (95% uncertainty interval €381.7 million to €431.5 million). Scenario and joint probabilistic analyses confirmed robustness under alternative utility values and surgical outcomes.

**Conclusion:**

Angiography-guided thyroidectomy may be a cost-saving innovation within publicly funded healthcare systems by reducing the incidence and chronic burden of postoperative hypoparathyroidism. These findings support broader adoption of fluorescence-guided surgery and underscore the need for real-world implementation studies to validate cost-effectiveness, and inform training and procurement strategies.

## Teaser

In a lifetime Markov model informed by contemporary surgical data, angiography-guided thyroidectomy was compared with conventional thyroidectomy for prevention of postoperative hypoparathyroidism. The intervention produced greater health benefits at lower overall cost, remaining cost-saving under extensive uncertainty analyses. Fluorescence-guided surgery may therefore represent a high-value intervention for publicly funded healthcare systems.

## Introduction

Total thyroidectomy is performed routinely for benign and malignant thyroid disease, with procedure volumes continuing to rise worldwide as indications expand and surgical safety improves. Postoperative hypoparathyroidism (hypoPT) remains the most frequent complication, arising from devascularization, trauma, or unintended excision of the parathyroid glands. Across large cohorts and national registries, transient hypoPT occurs in approximately 17–48% of patients^1–4^, whereas around 2–15% develop permanent hypoPT^1–7^. Rates vary with surgical volume and expertise, extent of surgery, and patient-related factors, and are consistently higher in lower-volume centres, when central neck dissection is performed, and among older patients or women^1,4,5,8,9^.

Permanent (chronic) postsurgical hypoPT is associated with substantial long-term morbidity. Patients are at increased risk of renal complications (including chronic kidney disease and nephrolithiasis), as well as neurological manifestations such as seizures and basal ganglia calcifications. These complications contribute to impaired health-related quality of life (HRQoL) compared with patients who have undergone thyroidectomy without hypoPT^3,10,11^. Beyond biochemical derangements, permanent hypoPT is characterized by persistent functional impairment and sustained healthcare utilization. Additionally, although conventional therapy can correct serum calcium biochemically, it does not restore physiological parathyroid hormone function. Therefore, many patients continue to experience a myriad of symptoms, such as fatigue, neurocognitive impairment, anxiety, and reduced functional capacity despite achieving biochemical control^8,9,12–14^. As a result, chronic hypoPT requires lifelong monitoring and treatment, generating a cumulative clinical and economic burden for both patients and health systems.

Indocyanine green (ICG) fluorescence angiography provides real-time appraisal of parathyroid perfusion during thyroidectomy and allows mapping of feeding vessels before dissection. In a previous study from the authors group^15^, in which all parathyroid glands (PGs) were identified, the presence of at least one well perfused gland on ICG angiography reliably predicted immediate postoperative serum calcium levels within the normal range. Building on this work, they subsequently developed the angiography-guided thyroidectomy (A-GT) technique, which uses ICG mapping before thyroid dissection to visualize the arterial supply to the PGs. This strategy allows preservation of the PGs and their nourishing vessels before proceeding with thyroidectomy. This approach not only significantly reduced both postoperative and permanent hypoPT overall, but also among patients undergoing conventional thyroidectomy (TT) with central neck dissection^16–18^.

Prevention of postoperative hypoPT is clinically meaningful, as even a modest reduction in permanent disease may yield substantial long-term benefits in terms of HRQoL, renal outcomes, and healthcare use^19^. From a surgical perspective, interventions that reduce permanent hypoPT without adding operative complexity or morbidity are particularly attractive. However, despite growing adoption of fluorescence-guided endocrine surgery, the cost-effectiveness of incorporating ICG angiography into routine TT has not been evaluated formally.

This study aimed to evaluate the cost-effectiveness of A-GT compared with TT from the perspective of a publicly funded health system, quantifying differences in postoperative hypoPT outcomes, health-related quality-adjusted life-years (QALYs), and healthcare costs.

## Methods

This study was based on modelling and secondary data analysis, with no involvement of human subjects or identifiable personal data; therefore, ethics committee approval was not required.

### Study design and setting

Throughout this manuscript, thyroidectomy refers specifically to total thyroidectomy, consistent with current English surgical nomenclature in which thyroidectomy denotes total resection and lobectomy denotes partial resection. A model-based cost-effectiveness analysis comparing A-GT with TT was conducted from the perspective of the Spanish National Health System (NHS), following National Institute for Health and Care Excellence (NICE) reference-case principles. Costs were valued in 2023 euros (€), and both costs and QALYs were discounted at 3.5% annually. The model simulated an open cohort of adults undergoing TT (with or without central neck dissection), with 1428 new patients annually, reflecting regional surgical activity. A 35-year horizon captured long-term monitoring and morbidity associated with chronic hypoPT, corresponding to the average remaining life expectancy of adults undergoing TT in Spain (approximately 85 years). This open-cohort design allowed modelling of the cumulative population-level burden and reflected the continuous inflow of patients seen in routine surgical care.

The analysis reflects Spanish NHS cost structures and surgical volume, but external generalizability may vary depending on local pricing, clinical practice, and technology adoption pathways.

### Model structure and assumptions

**Figure 1:**
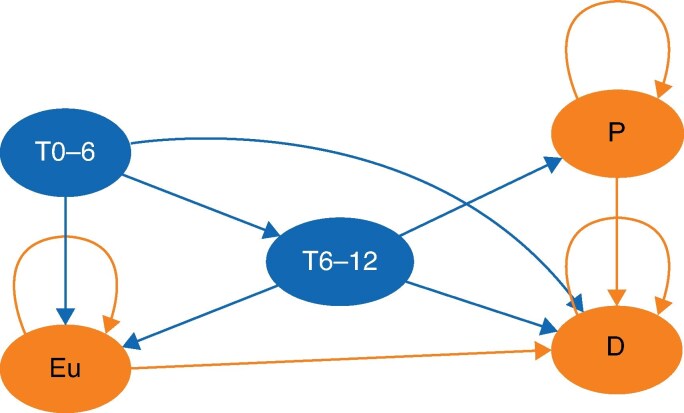
Markov model structure Patients enter the model after total thyroidectomy and can transition between mutually exclusive postoperative health states: euparathyroidism (Eu), transient hypoparathyroidism (T0–6), transient hypoparathyroidism (T6–12), permanent hypoparathyroidism (P), and death (D). Individuals who remain with hypoparathyroidism at 12 months are classified as having permanent hypoparathyroidism. All states carry background age- and sex-adjusted mortality risk, with an excess mortality hazard applied to permanent disease. Transitions occur in half-year cycles during the first postoperative year and annually thereafter. Dynamic transition probabilities for each cycle and year of follow-up are reported in *Tables S1–S21*.

A Markov model with a tunnel-state structure (splitting the transient state into sequential short-term phases to model recovery timing and allow accurate QALY and cost accrual) was used to represent postoperative parathyroid function (*Fig. 1*). Four mutually exclusive health states were modelled: euparathyroidism, transient hypoPT, permanent hypoPT, and death. Recovery from transient hypoPT was modelled in two phases: < 6 months and 6–12 months. Patients remaining in the transient hypoPT state after 12 months were classified as having permanent hypoPT, consistent with the 2022 International Task Force definition^20^. Reversal from permanent hypoPT was not permitted in the base case.

Background mortality was age- and sex-adjusted using national life-tables^21^, with excess mortality applied to permanent hypoPT using published hazard ratios^22^. The female : male case mix was pragmatically set at 70 : 30, reflecting a midpoint between published surgical cohorts^3,18,23^. Each cycle represented half-year intervals in year 1 and annual cycles thereafter, with a half-cycle correction.

### Model inputs

Baseline hypoPT risks after TT were sourced from a Spanish nationwide, multicentre cohort study^3^ capturing routine clinical practice across both high- and lower-volume hospitals, rather than outcomes from selected high-volume specialized centres alone. Corresponding rates following A-GT were informed by a comparative cohort study from the authors’ group^18^. Transition probabilities were calibrated iteratively by adjusting recovery parameters until modelled state occupancy aligned with observed prevalence over time; age-dependent parameters were updated every 5 years (*Tables S1–S21*). Full inputs are summarized in *Table 1*.

**Table 1:** Model inputs

	Value	Reference
**Cohort and epidemiology**		
Annual surgery intake (*n*)	1428	Spanish NHS^24^
Entry age (years)	50	Moreno Llorente *et al*.^18^
Female : male ratio undergoing TT	70 : 30	Assumed*
RR of hypoPT at discharge (female *versus* male)	5.7	Villarroya-Marquina *et al*.^23^
**Natural history and mortality**		
Excess mortality (HR) of permanent hypoPT *versus* EuPT	2.48	Reinke *et al*.^22^
5-year mortality multipliers by age (years)		
50–54	2.17	INE^21^
55–59	3.89	INE^21^
60–64	6.51	INE^21^
65–69	9.18	INE^21^
70–74	14.58	INE^21^
75–79	24.33	INE^21^
80–84	43.90	INE^21^
**Economic framework**		
Annual discount rate (%)	3.5	NICE
DRG mix (major : extreme)	90 : 10	Assumed
ER case-severity mix (mild–moderate : severe)	80 : 20	Assumed
Network for capital allocation (no. of tertiary centres)	9	Assumed
**Post-thyroidectomy hypoPT data**		
Prevalence after conventional thyroidectomy		
HypoPT at discharge (%)	51.7	Diez *et al*.^3^
Transient hypoPT (%)	22.9	Diez *et al*.^3^
Permanent hypoPT (%)	16.7	Diez *et al*.^3^
Risk ratio (A-GT *versus* TT)		
At discharge	0.222	Continuity-corrected from Moreno Llorente *et al*.^18^
Permanent hypoPT (A-GT *versus* TT)	0.096	Continuity-corrected from Moreno Llorente *et al*.^18^
**Cost items (unit cost, year 2023)**		
Surgical intake cost		
DRG major complexity (€)	16 326.00	CatSalut^25^
DRG extreme complexity (€)	39 128.02	CatSalut^25^
ICG-related incremental costs		
ICG vial (€)	37.16	CatSalut^25^
NIR camera system (€)	80 000.00	Stryker Iberia
Annual maintenance (NIR camera system) (€)	5% acquisition cost	Assumed
Personnel training (€)	7702.40	CatSalut^25^
ER care—acute hypocalcaemia		
DRG mild–moderate (€)	284.16	CatSalut^25^
DRG severe (€)	407.30	CatSalut^25^
Routine follow-up		
Outpatient clinic (first visit)—once in year 1 (€)	199.86	CatSalut^25^
Outpatient clinic (follow-up visit)—twice per year (€)	92.83	CatSalut^25^
Serum calcium measurement—twice per year (€)	0.85	CatSalut^25^
Serum phosphate measurement—twice per year (€)	0.77	CatSalut^25^
Serum albumin measurement—twice per year (€)	2.95	CatSalut^25^
Serum calcidiol measurement—once per year (€)	8.46	CatSalut^25^
Serum PTH measurement—once per year (€)	11.76	CatSalut^25^
HypoPT treatment costs†		
Calcium (1000 mg/day) + vitamin D3 (880 units/day) (€)	4.76	CatSalut^25^
Calcitriol (0.25 µg/day) (€)	2.79	CatSalut^25^
Hydrochlorothiazide (25 mg/day) (€)	1.40	CatSalut^25^
**Utilities (mapped EQ-5D^TM^)**		**Reference**
Base case		
EuPT	0.797	Jørgensen *et al*.^26^
Transient hypoPT	0.603	Jørgensen *et al*.^26^
Permanent hypoPT	0.603	Jørgensen *et al*.^26^
Death	0.000	–
Scenario 1		
EuPT	0.738	Cherchir *et al*.^27^
Transient hypoPT	0.508	Cherchir *et al*.^27^
Permanent hypoPT	0.508	Cherchir *et al*.^27^
Death	0.000	–
Scenario 2		
EuPT	0.808	Astor *et al*.^28^
Transient hypoPT	0.619	Astor *et al*.^28^
Permanent hypoPT	0.619	Astor *et al*.^28^
Death	0.000	–

^*^Owing to variability in reported female proportions across published series, a pragmatic midpoint of 0.70 was selected for the base case^3,18,23^. †Hypoparathyroidism (hypoPT) treatment costs represent a 60% payer share. NHS, National Health System; TT, conventional thyroidectomy; RR, risk ratio; HR, hazard ratio; EuPT, euparathyroidism; INE, Instituto Nacional de Estadística; NICE, National Institute for Health and Care Excellence; DRG, diagnosis-related group; ER, emergency room; A-GT, angiography-guided thyroidectomy; ICG, indocyanine green; NIR, near-infrared; PTH, parathyroid hormone.

### Costs

Costs were estimated from the Spanish NHS perspective. The ICG strategy included use of ICG vials and a near-infrared (NIR) fluorescence system (SPY-PHI; Stryker Endoscopy, San Jose, CA, USA). Capital and training costs were annualized over 10 years at 3.5% and allocated across a network of nine tertiary referral centres in Catalonia (*Tables S22* and *S23*), selected as high-volume institutions likely to adopt NIR imaging; no centre-specific clinical effects were modelled. Surgical intake costs used diagnosis-related group (DRG) tariffs from the Catalan Health Institute^25^, weighted for thyroidectomy complexity. Unit costs were updated to 2023 euros using the Consumer Price Index (*Table 1*).

### Effects

Health outcomes were expressed as QALYs, calculated as utility scores multiplied by time spent in each health state. Because direct EQ-5D™ (EuroQol, Rotterdam, the Netherlands) utilities for hypoPT are scarce, reported SF-36^®^ values were mapped using the algorithm validated by Rowen *et al*.^29^ (*Tables S24* and *S25*). Base-case utilities for euparathyroidism and chronic hypoPT were sourced from peer-reviewed studies reporting HRQoL in post-thyroidectomy cohorts^26^; no Spain-specific utility values were identified. Evidence does not consistently distinguish HRQoL differences between transient and permanent hypoPT; therefore, a common utility decrement was applied in the base case, with duration-specific alternatives tested in scenario analyses^27,28^.

### Base-case and sensitivity analyses

The base case compared A-GT with TT using a probabilistic framework (10 000 Monte-Carlo simulations) to propagate uncertainty around each strategy’s deterministic mean totals (costs, QALYs, net monetary benefits (NMBs)) via correlated γ draws linked by a Gaussian copula to reflect higher costs and lower QALYs with greater hypoPT time. Common random numbers preserved between-arm correlation; uncertainty was summarized with 95% probabilistic uncertainty intervals (UIs). The incremental cost-effectiveness ratio (ICER) was computed as ratio of means with Fieller 95% confidence intervals (appropriate when both numerator and denominator are stochastic). Cost-effectiveness was judged at a willingness-to-pay (WTP) threshold of €30 000 per QALY (Spanish decision-making reference value).

A deterministic one-way sensitivity analysis varied parameters by ±20% around base values; exceptions were the discount rate (±2%), the emergency-referral severity mix (base 70 : 30, tested at 60 : 40 and 80 : 20), and health-state utilities, which were varied by substituting published alternative value sets. For each level, the full model was re-solved deterministically.

A joint probabilistic sensitivity analysis (PSA) used the same stochastic structure as the base case, but varied key structural and clinical assumptions (recovery distribution, risk ratio of permanent hypoPT under ICG, excess-mortality hazard ratio, utility source, emergency-referral severity mix, discount rate, and annual intake).

In addition, a deterministic threshold analysis was conducted to identify the baseline permanent hypoPT rate after TT at which A-GT ceased to be cost-effective (incremental NMB 0) and cost-saving (incremental cost 0) at a WTP threshold of €30 000 per QALY.

A non-parametric bootstrap validation (10 000 resamples) was undertaken to assess the stability and sampling variability of mean incremental results.

All modelling and statistical analyses were performed using R version 4.3.2 (R Foundation for Statistical Computing, Vienna, Austria) on macOS^®^ (Apple Inc., Cupertino, CA, USA).

## Results

### Base-case analysis

**Figure 2:**
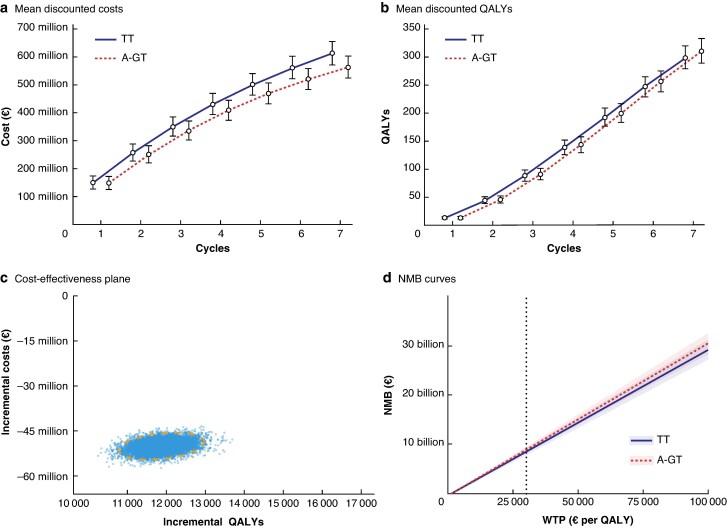
Cost and health-related quality-of-life outcomes for A-GT *versus* TT: base-case analysis **a** Mean cumulative discounted costs over the modelled time horizon, showing lower long-term health-system expenditure for angiography-guided thyroidectomy (A-GT) compared with conventional thyroidectomy (TT). **b** Mean cumulative discounted quality-adjusted life-years (QALYs), demonstrating greater lifetime health benefit with A-GT. **c** Cost-effectiveness plane for the base-case analysis—the orange dashed line represents the 95% confidence ellipse for the joint distribution of incremental costs and incremental QALYs. **d** Net monetary benefit (NMB) curves across willingness-to-pay (WTP) thresholds, showing consistently higher NMB for indocyanine green guidance; the dotted line denotes the Spanish reference threshold (€30 000 per QALY); shaded bands represent the 95% uncertainty intervals of NMB across the 10 000 PSA simulations at each WTP threshold; lines show the mean NMB.

In the base case, A-GT was both less costly and more effective than TT. Over the modelled lifetime horizon, mean total discounted costs were €563.7 million (95% UI €525.1 million to 603.5 million) for A-GT *versus* €613.2 million (€572.0 million to 656.0 million) for TT (*Fig. 2a*), corresponding to mean cost savings of €49.5 million with ICG. Health outcomes also favoured A-GT, with mean QALYs of 310 997 (95% UI 289 726 to 332 803) *versus* 299 107 (278 659 to 320 048) with TT (*Fig. 2b*), yielding a mean gain of 11 889 QALYs.

A-GT therefore dominated TT, yielding lower costs and greater QALYs (*Fig. 2c*). The ICER was −€4167 (95% confidence interval −€4171 to −€4162) per QALY gained, indicating cost savings alongside improved health outcomes. At a WTP threshold of €30 000 per QALY, the incremental NMB was €406.2 million (95% UI €381.7 million to 431.5 million), consistent with the NMB WTP profile (*Fig. 2d*). The breakeven activity was approximately 147 thyroidectomies per centre per year, above which A-GT yields non-negative NMBs.

A complete breakdown by 5-year horizons and the lifetime totals of costs, QALYs, ICER, and NMBs is provided in *Tables S26–S35* and *S36–S41*, respectively.

### Sensitivity analyses

Two alternative health-state utility mappings were examined to test the robustness of the base-case results to different HRQoL estimates (*Tables S24* and *S25*). As expected, costs are invariant to the choice of utility set.

**Figure 3:**
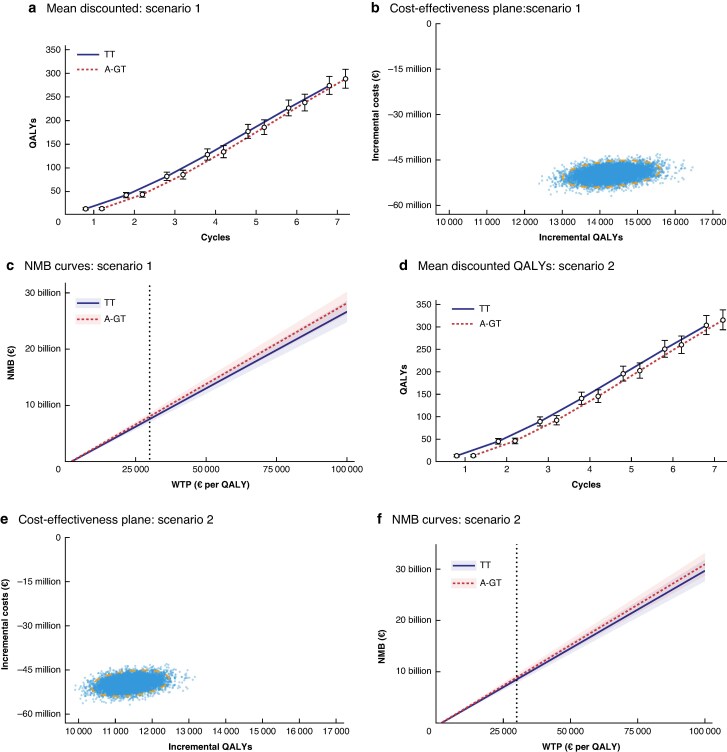
Cost-effectiveness outcomes for A-GT versus TT under alternative utility scenarios (Scenarios 1 and 2) **a** Mean cumulative discounted quality-adjusted life-years (QALYs) over the modelled time horizon for scenario 1, showing lower long-term health-system expenditure with angiography-guided thyroidectomy (A-GT) compared with conventional thyroidectomy (TT). **b** Cost-effectiveness plane for scenario 1, displaying 10 000 probabilistic simulations of incremental costs and incremental QALYs. All points lie in the southeast quadrant, indicating lower lifetime costs and greater health benefit with A-GT. The dashed ellipse represents the 95% normal confidence region for the joint distribution. **c** Net monetary benefit (NMB) curves for scenario 1 across willingness-to-pay (WTP) thresholds. A-GT consistently yields higher NMB, at the threshold of €30 000 per QALY (dotted); A-GT is economically dominant. **d** Mean cumulative discounted QALYs for scenario 2 (higher-utility set), again demonstrating greater health benefit with A-GT across the full time horizon. **e** Cost-effectiveness plane for scenario 2, with all simulations located in the southeast quadrant, confirming persistent dominance under more favourable health-related quality-of-life assumptions. The dashed ellipse denotes the 95% normal confidence region. **f** NMB curves for scenario 2, showing sustained economic superiority of A-GT across all WTP thresholds examined.

In scenario 1, utilities were lower overall. Total discounted QALYs were 287 797 for A-GT and 273 509 for TT, yielding 14 288 incremental QALYs. ICG remained dominant—producing higher QALYs at lower lifetime cost—with an ICER of −€3467 per QALY and a mean incremental NMB of €478.2 million at a threshold of €30 000 per QALY (*Fig. 3a–c* and *Table S41*).

In Scenario 2, utilities were higher overall. Total discounted QALYs were 315 066 for A-GT and 303 529 for TT, corresponding to 11 537 incremental QALYs. A-GT again dominated TT, with an ICER of −€4294 per QALY and a mean incremental NMB of €395.6 million (*Fig. 3d–f* and *Table S41*).

**Figure 4:**
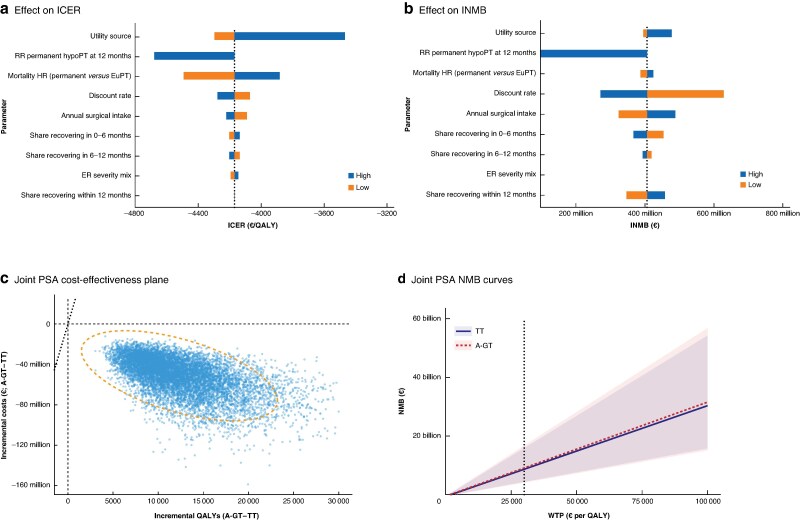
Deterministic and probabilistic sensitivity analyses **a** One-way sensitivity analysis showing the effect of varying key clinical and structural parameters on the incremental cost-effectiveness ratio (ICER) for angiography-guided thyroidectomy (A-GT) *versus* conventional thyroidectomy (TT). Bars represent low and high values for each parameter; the vertical dotted line indicates the base-case ICER. **b** Corresponding effects on incremental net monetary benefit (INMB) at a willingness-to-pay (WTP) threshold of €30 000 per QALY. A-GT remained economically dominant—improving health outcomes at lower total cost—across all parameter ranges. **c** Joint probabilistic sensitivity analysis (PSA) cost-effectiveness plane for the structural-uncertainty scenario set, illustrating wider dispersion than in the base case owing to simultaneous variation of multiple model assumptions. Grey dashed lines mark the reference axes of the cost-effectiveness plane (incremental QALYs = 0 and incremental costs = 0); the red dashed line indicates the €30 000 per QALY WTP threshold. The dashed ellipse denotes the 95% normal confidence region for the joint distribution of incremental costs and QALYs. **d** Net monetary benefit (NMB) curves from the joint PSA, showing consistently higher NMB for A-GT across all WTP thresholds. The dashed vertical line marks the Spanish reference threshold (€30 000 per QALY). RR, risk ratio; hypoPT, hypoparathyroidism; HR, hazard ratio; EuPT, euparathyroidism; ER, emergency room.

In the deterministic one-way sensitivity analysis evaluated at a coefficient of variation (CV) = 0.20, the incremental NMB at a threshold of €30 000 per QALY was most influenced by the annual discount rate, annual surgical intake, and the overall share recovering by 12 months. Although variation in these parameters shifted the incremental NMB, dominance of A-GT was never overturned (*Fig. 4a,b* and *Table S42*). Results were consistent under alternative dispersion assumptions (CV = 0.10 and 0.30), with the same parameters ranking as the most influential drivers of incremental NMB (*Table S42* and *Figs S1–S4*).

When all key model parameters were varied simultaneously (joint PSA), A-GT remained the dominant strategy. Across 10 000 probabilistic simulations, mean incremental cost was −€51.5 million, mean incremental QALYs were 12 386, and the resulting ICER was −€4155 per QALY gained (*Fig. 4c*). As expected, the joint PSA cost-effectiveness plane exhibited substantially greater dispersion than the base case because structural and epidemiological assumptions were varied alongside stochastic uncertainty in costs and QALYs. This widened the range of plausible incremental costs and incremental QALYs, producing a more diffuse distribution of simulations. The mean incremental NMB was €423.0 million (95% UI €209.3 million to €769.5 million) at a WTP threshold of €30 000 per QALY. All simulations fell in the southeast quadrant of the cost-effectiveness plane, and the NMB curve for A-GT lay above that of TT across all thresholds (*Fig. 4d*).

In an additional threshold analysis varying the baseline permanent hypoPT rate after TT at 12 months, the incremental NMB remained positive for rates ≥ 0.6%. A-GT also became cost-saving above approximately 0.6% and was dominant across all clinically plausible permanent hypoPT rates examined (0.5–20.0%).

Bootstrap resampling of the base-case simulations (10 000 resamples) confirmed the stability of the incremental results (*Table S43*). The distributions of incremental costs, QALYs, NMB, and the ICER were tightly centred around the means (*Figs S5–S8*), and A-GT remained dominant in all resamples. No resample reversed the direction of effect, indicating that the findings were not driven by outlier simulations or distributional assumptions.

## Discussion

In this model-based economic evaluation informed by contemporary surgical practice, A-GT was associated with lower lifetime healthcare costs and increased QALYs compared with TT. This direction of effect was consistent across deterministic, probabilistic, and scenario-based analyses, indicating that the findings were resilient to uncertainty in clinical, economic, and structural assumptions. The observed economic advantage arises primarily from reductions in postoperative hypoPT, which in turn decrease long-term monitoring requirements, supplementation needs, and downstream complications. Although the magnitude of cost savings and QALY gains varied depending on model assumptions, the overall conclusion—namely, that A-GT is likely to be cost-saving while improving patient outcomes—was consistent. To the authors’ knowledge, this is the first study to quantify the cost-effectiveness of ICG angiography for parathyroid preservation. A-GT appears to offer good value for money within the Spanish NHS by reducing the incidence and long-term burden of postoperative hypoPT. These findings support structured training, standardized adoption across surgical centres, and future evaluation through multicentre implementation studies. Policy decisions should remain sensitive to local cost structures and clinical practices, and continued prospective monitoring is warranted to confirm whether anticipated reductions in chronic disease burden are realized in routine care.

This analysis builds directly on a multiyear research programme by the authors’ group aimed at refining, validating, and operationalizing A-GT to mitigate postoperative hypoPT. Whereas their previous studies^15–18^ focused on describing the surgical technique, and reporting its clinical rationale and outcomes, the present work aimed to assess whether those reductions translate into meaningful health-system value.

The present results are consistent with emerging evidence suggesting that real-time perfusion imaging may improve parathyroid preservation by enabling intraoperative identification of well vascularized glands^18,30,31^. A 2020 meta-analysis^32^ reported that NIR imaging, whether using autofluorescence or ICG, reduces short- and medium-term hypocalcaemia rates after TT. More recent studies^18,33–36^ specifically support the benefits of ICG angiography, suggesting improved gland preservation and reduced early complications. These clinical effects are particularly relevant given that permanent hypoPT is driven largely by devascularization injuries sustained at the time of surgery. Practical advantages, such as ease of integration and minimal training requirements^31,37^, further support its adoption.

Despite increasing clinical uptake, formal economic evaluations of NIR imaging remain scarce. The only comparable evaluation identified was a 2025 Australian model assessing NIR autofluorescence—but not A-GT—reporting an ICER above the nationally accepted threshold; autofluorescence was cost-effective only when the rate of permanent hypoPT exceeded 5%^38^. In contrast, A-GT in the present setting was not only cost-effective but cost-saving under all modelling scenarios. Several clinical and methodological features likely explain these different findings. Clinically, NIR autofluorescence enhances parathyroid gland identification but does not assess perfusion, whereas A-GT visualizes the arterial supply in real time and may better prevent the devascularization injuries that drive permanent hypoPT^31,34,36,37^. Because permanent hypoPT is associated with substantial lifelong costs and HRQoL burden, technologies that more effectively reduce its incidence are expected to yield proportionally greater long-term economic benefit. Accordingly, the low permanent hypoPT threshold (< 1%) identified in the present analysis should be interpreted as an indicator of robustness rather than an epidemiological expectation, reinforcing that the economic conclusions are unlikely to be sensitive to variation in baseline complication rates observed in routine practice.

Methodologically, the Australian model used a decision-analytic structure with 5.0% discounting, which places less weight on delayed complications. The present state-transition, open-cohort Markov model with 3.5% discounting explicitly captures the full downstream burden of chronic hypoPT complications and the accumulation of affected patients at the population level over time. Additionally, capital costs were further amortized across a regional centre network, reducing per-patient costs in high-volume contexts. These combined clinical and methodological differences likely contributed to the more favourable economic profile observed for A-GT.

Beyond ICERs, the breakeven offers important practical insights for decision-makers. A-GT became cost-saving at approximately 147 thyroidectomies per year, an attainable threshold in high-volume centres or regional networks, but also informative for lower-volume institutions. Importantly, evidence shows that postoperative hypoPT rates are strongly volume- and context-dependent, varying with surgeon experience, case mix, extent of surgery, and institutional protocols. For instance, a Danish retrospective cohort study^1^ reported a 31.5% permanent hypoPT rate in a low-volume, non-parathyroid centre, far exceeding rates observed in specialized units. Accordingly, the relatively high base-case hypoPT rates used in this model (51.7% hypoPT at discharge and 16.7% permanent) should be interpreted as reflecting nationwide real-world practice across mixed-volume centres^3^, rather than benchmark outcomes from high-volume expert units. In this context, centres with lower procedural volumes or limited parathyroid-specific expertise may derive proportionally greater benefit from A-GT, as preventing a single case of permanent hypoPT averts substantial lifelong cost and morbidity. Moreover, the magnitude of population-level QALY gains highlights the relevance of even modest per-patient benefits when scaled to routine surgical practice—corresponding to a cumulative gain of nearly 12 000 years of life lived in full health across the modelled cohort over 35 years. Thus, although the breakeven threshold reflects a conservative estimate based on average Spanish outcomes, A-GT may be particularly advantageous in settings with a higher baseline complication risk.

Notably, health-system contextual factors may also have shaped the present results. Public-sector pricing, DRG-based reimbursement, and centralized procurement (regional tendering) likely reduce capital and operating device costs^39^. Additionally, the relatively high burden of postoperative hypoPT in Spain may also enhance the marginal value of prevention-focused interventions such as A-GT. These considerations underscore the importance of tailoring economic evaluations to local health-system structures, surgical volumes, and practice patterns, and caution against extrapolating ICERs across jurisdictions without recalibration.

Although other prospective studies^18,31,40^ support the clinical value of ICG , none have systematically examined whether reductions in hypoPT translate into favourable cost-effectiveness profiles. This gap reflects a broader challenge in the surgical field, where the economic value of innovation is often underexplored, despite growing evidence that many essential surgical procedures are cost-effective across diverse global contexts^41,42^. In the present model, much of the benefit accrued from preventing permanent hypoPT, translating into a widening divergence in cumulative costs and QALYs over time. In probabilistic analyses, virtually all simulations fell in the southeast quadrant, indicating lower costs and greater effectiveness under joint uncertainty.

Structured prioritization frameworks have been proposed to support early adoption decisions for surgical innovations by helping stakeholders balance clinical promise, cost implications, and implementation feasibility^43^. For surgeons, ICG fluorescence is a relatively low-disruption addition to operative workflow that may enhance parathyroid preservation, particularly in training environments, complex cases, and centres with variable thyroidectomy volume. For hospital managers and procurement bodies, the breakeven analysis suggests that capital costs are more readily amortized when systems are shared across high-volume centres or integrated into regional surgical networks^44^. For health-system decision-makers, the results indicate that adopting ICG guidance could align with goals of reducing avoidable chronic complications and long-term care expenditure. Nonetheless, implementation decisions must account for opportunity costs, local surgical capacity, patient priorities, and procedure learning curve, which could increase operative time modestly. Notably, although the learning curve was not modelled explicitly, it is likely to diminish rapidly with technical familiarity.

Generalizability to other publicly funded health systems should be approached with caution. Cost-effectiveness results are inherently context-dependent, shaped by local health-system structure, pricing mechanisms, surgical infrastructure, and case-mix characteristics. Variability in healthcare resource availability, institutional cost accounting practices, and capital procurement pathways means that direct transfer of cost estimates or ICERs may be misleading. Furthermore, methodological heterogeneity across economic evaluations, such as differences in costing approaches, outcome metrics, and time horizons, limits comparability across settings. These challenges are well documented in the global surgical cost-effectiveness literature, highlighting the difficulty of extrapolating model-based results across jurisdictions without adjustment^41^. Additionally, patient demographics, disease burden, and baseline complication risks can influence both costs and effectiveness, particularly in countries with different thyroidectomy volumes or training pathways. Recalibration using country-specific epidemiology, costing, and practice patterns is therefore essential^45^.

Consistent with this context dependency, the use of Spanish DRG tariffs and public-sector pricing in this study reflects the local financing landscape but may not align with reimbursement models or resource use patterns elsewhere. In particular, health systems operating under fee-for-service reimbursement, private insurance arrangements, or hospital-level capital financing may experience different cost offsets and adoption incentives than those modelled here. Under fee-for-service models, reductions in postoperative complications may not translate directly into financial savings for providers, even when they yield efficiency gains at health system level. Conversely, systems in which hospitals bear direct responsibility for long-term complication management or capital investment may place greater value on interventions that prevent chronic morbidity. Differences in how capital equipment costs are financed, depreciated, or reimbursed, such as through bundled payments, activity-based funding, or separate capital budgets, may further influence the apparent cost-effectiveness of A-GT. These considerations underscore that, although the clinical mechanisms underpinning its benefits are likely to be generalizable, the magnitude and distribution of economic benefits will depend on local reimbursement structures and incentive alignment.

Several limitations should be acknowledged. Many clinical inputs were derived from observational rather than randomized data, introducing potential bias and confounding. The real-world effectiveness of A-GT may vary with surgeon experience, training conditions, patient mix, and adherence to technique. Health-state utilities were mapped from generic HRQoL instruments rather than elicited from Spanish patients, reflecting a limited and heterogeneous evidence base for hypoPT-specific utilities. Although alternative utility mappings and scenario analyses did not alter the direction of results, residual uncertainty in absolute QALY estimates cannot be excluded. The model assumed no reversal from permanent hypoPT and relied on limited evidence for late renal and neurological complications, requiring assumptions for long-term projections.

Costs were estimated using Spanish DRG tariffs and public-sector procurement prices, with capital equipment and training costs annualized over a 10-year device lifespan and allocated across a regional network of centres. Although similar procurement structures operate across most regions, these values may not reflect pricing or resource-use patterns in other health systems. Consequently, cost-effectiveness results should not be extrapolated without recalibration to local epidemiology, pricing, and surgical-volume patterns. Prospective multicentre data collection would strengthen the evidence base and refine long-term estimates.

## Supplementary Material

zrag095_Supplementary_Data

## Data Availability

The data underlying this article will be shared upon reasonable request to the corresponding author.
