# Supplementary material for: Cost-effectiveness of angiography-guided thyroidectomy to prevent postoperative hypoparathyroidism: Spanish National Health System model-based analysis
Source: BJS Open. 2026 Jul 22;10(4):zrag095. doi: 10.1093/bjsopen/zrag095 (PMC13390998; doi:10.1093/bjsopen/zrag095)
Supplement: zrag095_Supplementary_Data [file zrag095_supplementary_data.docx]

**Cost-Effectiveness of Indocyanine Green Angiography for Parathyroid Preservation in Total Thyroidectomy: A Model-Based Economic Evaluation**

Pablo Moreno-Llorente ^1, 2, *^, Marta Ruiz ^1, *^, Arantxa García-Barrasa ^1^, Marta Recasens-Subias ^1^, Itziar Larrañaga Barrera ^1^, Thiago Carnaval ^3, 4, 5^, Sebastián Videla ^4, 5, 6^

1. Unidad de Cirugía Endocrina, Hospital Universitari de Bellvitge, C/ Feixa Llarga s/n, L'Hospitalet de Llobregat, Barcelona, E-0897, Spain. 25108pml@gmail.com.
2. Departament de Ciències Clíniques, Facultat de Medicina i Ciències de la Salut, Universitat de Barcelona, Barcelona, Spain.
3. Methodological and Statistical Support Department, Fundació de Recerca Sant Joan de Déu, Esplugues de Llobregat, Barcelona, Spain.
4. Pharmacology Unit, Department of Pathology and Experimental Therapeutics, School of Medicine and Health Sciences, Institute of Neurosciences, University of Barcelona, L'Hospitalet de Llobregat, Spain.
5. Neuropharmacology & Pain Group, Neuroscience Program, Bellvitge Biomedical Research Institute, L'Hospitalet de Llobregat, Barcelona, Spain.
6. Clinical Research Support Area, Clinical Pharmacology Department, Germans Tries I Pujol University Hospital, Badalona, Spain.

^*^ These authors contributed equally and share first co-authorship.

**Corresponding authors:** Pablo Moreno-Llorente ([25108pml@gmail.com](mailto:25108pml@gmail.com); ORCID ID: 0000-0002-0718-2797), Marta Ruiz ([mruiz.65@alumni.unav.es](mailto:mruiz.65@alumni.unav.es))

**Supplementary Materials - Index**

| **Supplementary Methods** |  |
| --- | --- |
| Transition Probabilities | *2* |
| Estimation of Training Costs | *6* |
| Mapped QALYs | *7* |
| **Supplementary Results** |  |
| Means Breakdown by 5-Year Horizons | *8* |
| Mean Lifetime Totals Derived from Monte Carlo Simulations | *12* |
| Deterministic One-Way Sensitivity Analysis | *13* |
| Bootstrap Validation | *17* |
| **References** | *20* |

**Supplementary Methods**

**Transition Probabilities**

State-to-state transition probabilities for each arm (Conventional and ICG) were derived for early recovery (0–6 months), intermediate recovery (6–12 months), and subsequent annual transitions, incorporating attained-age background mortality and excess mortality in the permanent state. Because the model uses a tunnel-state structure for transient hypoPT, transition probabilities differ between the first (H1) and second (H2) half-cycles of the first year. From year 2 onward, transitions are modelled using annual matrices composed as the product of H1 × H2.

Transition probability matrices vary by attained age group, aligned with INE 5-year bands (50–54, 55–59, 60–64, etc.). They are piecewise constant within each band and update when the cohort crosses into the next band, based on attained age rather than calendar year.

1. H1 (Years 0 – 4)

| **From/To** | **Conventional** | | | | **ICG** | | | |
| --- | --- | --- | --- | --- | --- | --- | --- | --- |
|  | **Eu** | **T** | **P** | **D** | **Eu** | **T** | **P** | **D** |
| Eu | 0.999023 | 0.000000 | 0.000000 | 0.000977 | 0.999023 | 0.000000 | 0.000000 | 0.000977 |
| T | 0.525366 | 0.473657 | 0.000000 | 0.000977 | 0.525366 | 0.473657 | 0.000000 | 0.000977 |
| P | 0.000000 | 0.000000 | 0.999023 | 0.000977 | 0.000000 | 0.000000 | 0.999023 | 0.000977 |
| D | 0.000000 | 0.000000 | 0.000000 | 1.000000 | 0.000000 | 0.000000 | 0.000000 | 1.000000 |

1. H1 (Years 5 – 9)

| **From/To** | **Conventional** | | | | **ICG** | | | |
| --- | --- | --- | --- | --- | --- | --- | --- | --- |
|  | **Eu** | **T** | **P** | **D** | **Eu** | **T** | **P** | **D** |
| Eu | 0.998278 | 0.000000 | 0.000000 | 0.001722 | 0.998278 | 0.000000 | 0.000000 | 0.001722 |
| T | 0.524974 | 0.473304 | 0.000000 | 0.001722 | 0.524974 | 0.473304 | 0.000000 | 0.001722 |
| P | 0.000000 | 0.000000 | 0.998278 | 0.001722 | 0.000000 | 0.000000 | 0.998278 | 0.001722 |
| D | 0.000000 | 0.000000 | 0.000000 | 1.000000 | 0.000000 | 0.000000 | 0.000000 | 1.000000 |

1. H1 (Years 10 – 14)

| **From/To** | **Conventional** | | | | **ICG** | | | |
| --- | --- | --- | --- | --- | --- | --- | --- | --- |
|  | **Eu** | **T** | **P** | **D** | **Eu** | **T** | **P** | **D** |
| Eu | 0.997191 | 0.000000 | 0.000000 | 0.002809 | 0.997191 | 0.000000 | 0.000000 | 0.002809 |
| T | 0.524403 | 0.472788 | 0.000000 | 0.002809 | 0.524403 | 0.472788 | 0.000000 | 0.002809 |
| P | 0.000000 | 0.000000 | 0.997191 | 0.002809 | 0.000000 | 0.000000 | 0.997191 | 0.002809 |
| D | 0.000000 | 0.000000 | 0.000000 | 1.000000 | 0.000000 | 0.000000 | 0.000000 | 1.000000 |

1. H1 (Years 15 – 19)

| **From/To** | **Conventional** | | | | **ICG** | | | |
| --- | --- | --- | --- | --- | --- | --- | --- | --- |
|  | **Eu** | **T** | **P** | **D** | **Eu** | **T** | **P** | **D** |
| Eu | 0.996031 | 0.000000 | 0.000000 | 0.003969 | 0.996031 | 0.000000 | 0.000000 | 0.003969 |
| T | 0.523792 | 0.472238 | 0.000000 | 0.003969 | 0.523792 | 0.472238 | 0.000000 | 0.003969 |
| P | 0.000000 | 0.000000 | 0.996031 | 0.003969 | 0.000000 | 0.000000 | 0.996031 | 0.003969 |
| D | 0.000000 | 0.000000 | 0.000000 | 1.000000 | 0.000000 | 0.000000 | 0.000000 | 1.000000 |

1. H1 (Years 20 – 24)

| **From/To** | **Conventional** | | | | **ICG** | | | |
| --- | --- | --- | --- | --- | --- | --- | --- | --- |
|  | **Eu** | **T** | **P** | **D** | **Eu** | **T** | **P** | **D** |
| Eu | 0.993618 | 0.000000 | 0.000000 | 0.006382 | 0.993618 | 0.000000 | 0.000000 | 0.006382 |
| T | 0.522524 | 0.471094 | 0.000000 | 0.006382 | 0.522524 | 0.471094 | 0.000000 | 0.006382 |
| P | 0.000000 | 0.000000 | 0.993618 | 0.006382 | 0.000000 | 0.000000 | 0.993618 | 0.006382 |
| D | 0.000000 | 0.000000 | 0.000000 | 1.000000 | 0.000000 | 0.000000 | 0.000000 | 1.000000 |

1. H1 (Years 25 – 29)

| **From/To** | **Conventional** | | | | **ICG** | | | |
| --- | --- | --- | --- | --- | --- | --- | --- | --- |
|  | **Eu** | **T** | **P** | **D** | **Eu** | **T** | **P** | **D** |
| Eu | 0.989088 | 0.000000 | 0.000000 | 0.010912 | 0.989088 | 0.000000 | 0.000000 | 0.010912 |
| T | 0.520142 | 0.468947 | 0.000000 | 0.010912 | 0.520142 | 0.468947 | 0.000000 | 0.010912 |
| P | 0.000000 | 0.000000 | 0.989088 | 0.010912 | 0.000000 | 0.000000 | 0.989088 | 0.010912 |
| D | 0.000000 | 0.000000 | 0.000000 | 1.000000 | 0.000000 | 0.000000 | 0.000000 | 1.000000 |

1. H1 (Years 30 – 35)

| **From/To** | **Conventional** | | | | **ICG** | | | |
| --- | --- | --- | --- | --- | --- | --- | --- | --- |
|  | **Eu** | **T** | **P** | **D** | **Eu** | **T** | **P** | **D** |
| Eu | 0.979693 | 0.000000 | 0.000000 | 0.020307 | 0.979693 | 0.000000 | 0.000000 | 0.020307 |
| T | 0.515201 | 0.464492 | 0.000000 | 0.020307 | 0.515201 | 0.464492 | 0.000000 | 0.020307 |
| P | 0.000000 | 0.000000 | 0.979693 | 0.020307 | 0.000000 | 0.000000 | 0.979693 | 0.020307 |
| D | 0.000000 | 0.000000 | 0.000000 | 1.000000 | 0.000000 | 0.000000 | 0.000000 | 1.000000 |

1. H2 (Years 0 – 4)

| **From/To** | **Conventional** | | | | **ICG** | | | |
| --- | --- | --- | --- | --- | --- | --- | --- | --- |
|  | **Eu** | **T** | **P** | **D** | **Eu** | **T** | **P** | **D** |
| Eu | 0.999023 | 0.000000 | 0.000000 | 0.000977 | 0.999023 | 0.000000 | 0.000000 | 0.000977 |
| T | 0.270478 | 0.000000 | 0.728545 | 0.000977 | 0.928971 | 0.000000 | 0.070052 | 0.000977 |
| P | 0.000000 | 0.000000 | 0.997579 | 0.002421 | 0.000000 | 0.000000 | 0.997579 | 0.002421 |
| D | 0.000000 | 0.000000 | 0.000000 | 1.000000 | 0.000000 | 0.000000 | 0.000000 | 1.000000 |

1. H2 (Years 5 – 9)

| **From/To** | **Conventional** | | | | **ICG** | | | |
| --- | --- | --- | --- | --- | --- | --- | --- | --- |
|  | **Eu** | **T** | **P** | **D** | **Eu** | **T** | **P** | **D** |
| Eu | 0.998278 | 0.000000 | 0.000000 | 0.001722 | 0.998278 | 0.000000 | 0.000000 | 0.001722 |
| T | 0.270276 | 0.000000 | 0.728002 | 0.001722 | 0.928278 | 0.000000 | 0.070000 | 0.001722 |
| P | 0.000000 | 0.000000 | 0.995736 | 0.004264 | 0.000000 | 0.000000 | 0.995736 | 0.004264 |
| D | 0.000000 | 0.000000 | 0.000000 | 1.000000 | 0.000000 | 0.000000 | 0.000000 | 1.000000 |

1. H2 (Years 10 – 14)

| **From/To** | **Conventional** | | | | **ICG** | | | |
| --- | --- | --- | --- | --- | --- | --- | --- | --- |
|  | **Eu** | **T** | **P** | **D** | **Eu** | **T** | **P** | **D** |
| Eu | 0.997191 | 0.000000 | 0.000000 | 0.002809 | 0.997191 | 0.000000 | 0.000000 | 0.002809 |
| T | 0.269982 | 0.000000 | 0.727209 | 0.002809 | 0.927267 | 0.000000 | 0.069924 | 0.002809 |
| P | 0.000000 | 0.000000 | 0.993049 | 0.006951 | 0.000000 | 0.000000 | 0.993049 | 0.006951 |
| D | 0.000000 | 0.000000 | 0.000000 | 1.000000 | 0.000000 | 0.000000 | 0.000000 | 1.000000 |

1. H2 (Years 15 – 19)

| **From/To** | **Conventional** | | | | **ICG** | | | |
| --- | --- | --- | --- | --- | --- | --- | --- | --- |
|  | **Eu** | **T** | **P** | **D** | **Eu** | **T** | **P** | **D** |
| Eu | 0.996031 | 0.000000 | 0.000000 | 0.003969 | 0.996031 | 0.000000 | 0.000000 | 0.003969 |
| T | 0.269668 | 0.000000 | 0.726363 | 0.003969 | 0.926188 | 0.000000 | 0.069843 | 0.003969 |
| P | 0.000000 | 0.000000 | 0.990185 | 0.009815 | 0.000000 | 0.000000 | 0.990185 | 0.009815 |
| D | 0.000000 | 0.000000 | 0.000000 | 1.000000 | 0.000000 | 0.000000 | 0.000000 | 1.000000 |

1. H2 (Years 20 – 24)

| **From/To** | **Conventional** | | | | **ICG** | | | |
| --- | --- | --- | --- | --- | --- | --- | --- | --- |
|  | **Eu** | **T** | **P** | **D** | **Eu** | **T** | **P** | **D** |
| Eu | 0.993618 | 0.000000 | 0.000000 | 0.006382 | 0.993618 | 0.000000 | 0.000000 | 0.006382 |
| T | 0.269015 | 0.000000 | 0.724604 | 0.006382 | 0.923945 | 0.000000 | 0.069673 | 0.006382 |
| P | 0.000000 | 0.000000 | 0.984248 | 0.015752 | 0.000000 | 0.000000 | 0.984248 | 0.015752 |
| D | 0.000000 | 0.000000 | 0.000000 | 1.000000 | 0.000000 | 0.000000 | 0.000000 | 1.000000 |

1. H2 (Years 25 – 29)

| **From/To** | **Conventional** | | | | **ICG** | | | |
| --- | --- | --- | --- | --- | --- | --- | --- | --- |
|  | **Eu** | **T** | **P** | **D** | **Eu** | **T** | **P** | **D** |
| Eu | 0.989088 | 0.000000 | 0.000000 | 0.010912 | 0.989088 | 0.000000 | 0.000000 | 0.010912 |
| T | 0.267788 | 0.000000 | 0.721300 | 0.010912 | 0.919732 | 0.000000 | 0.069356 | 0.010912 |
| P | 0.000000 | 0.000000 | 0.973157 | 0.026843 | 0.000000 | 0.000000 | 0.973157 | 0.026843 |
| D | 0.000000 | 0.000000 | 0.000000 | 1.000000 | 0.000000 | 0.000000 | 0.000000 | 1.000000 |

1. H2 (Years 30 – 35)

| **From/To** | **Conventional** | | | | **ICG** | | | |
| --- | --- | --- | --- | --- | --- | --- | --- | --- |
|  | **Eu** | **T** | **P** | **D** | **Eu** | **T** | **P** | **D** |
| Eu | 0.979693 | 0.000000 | 0.000000 | 0.020307 | 0.979693 | 0.000000 | 0.000000 | 0.020307 |
| T | 0.265244 | 0.000000 | 0.714448 | 0.020307 | 0.910996 | 0.000000 | 0.068697 | 0.020307 |
| P | 0.000000 | 0.000000 | 0.950392 | 0.049608 | 0.000000 | 0.000000 | 0.950392 | 0.049608 |
| D | 0.000000 | 0.000000 | 0.000000 | 1.000000 | 0.000000 | 0.000000 | 0.000000 | 1.000000 |

1. Annual (Years 0 – 4)

| **From/To** | **Conventional** | | | | **ICG** | | | |
| --- | --- | --- | --- | --- | --- | --- | --- | --- |
|  | **Eu** | **T** | **P** | **D** | **Eu** | **T** | **P** | **D** |
| Eu | 0.998047 | 0.000000 | 0.000000 | 0.001953 | 0.998047 | 0.000000 | 0.000000 | 0.001953 |
| T | 0.652967 | 0.000000 | 0.345080 | 0.001953 | 0.964866 | 0.000000 | 0.033181 | 0.001953 |
| P | 0.000000 | 0.000000 | 0.996604 | 0.003396 | 0.000000 | 0.000000 | 0.996604 | 0.003396 |
| D | 0.000000 | 0.000000 | 0.000000 | 1.000000 | 0.000000 | 0.000000 | 0.000000 | 1.000000 |

1. Annual (Years 5 – 9)

| **From/To** | **Conventional** | | | | **ICG** | | | |
| --- | --- | --- | --- | --- | --- | --- | --- | --- |
|  | **Eu** | **T** | **P** | **D** | **Eu** | **T** | **P** | **D** |
| Eu | 0.996559 | 0.000000 | 0.000000 | 0.003441 | 0.996559 | 0.000000 | 0.000000 | 0.003441 |
| T | 0.651993 | 0.000000 | 0.344566 | 0.003441 | 0.963428 | 0.000000 | 0.033131 | 0.003441 |
| P | 0.000000 | 0.000000 | 0.994021 | 0.005979 | 0.000000 | 0.000000 | 0.994021 | 0.005979 |
| D | 0.000000 | 0.000000 | 0.000000 | 1.000000 | 0.000000 | 0.000000 | 0.000000 | 1.000000 |

1. Annual (Years 10 – 14)

| **From/To** | **Conventional** | | | | **ICG** | | | |
| --- | --- | --- | --- | --- | --- | --- | --- | --- |
|  | **Eu** | **T** | **P** | **D** | **Eu** | **T** | **P** | **D** |
| Eu | 0.994390 | 0.000000 | 0.000000 | 0.005610 | 0.994390 | 0.000000 | 0.000000 | 0.005610 |
| T | 0.650574 | 0.000000 | 0.343816 | 0.005610 | 0.961331 | 0.000000 | 0.033059 | 0.005610 |
| P | 0.000000 | 0.000000 | 0.990259 | 0.009741 | 0.000000 | 0.000000 | 0.990259 | 0.009741 |
| D | 0.000000 | 0.000000 | 0.000000 | 1.000000 | 0.000000 | 0.000000 | 0.000000 | 1.000000 |

1. Annual (Years 15 – 19)

| **From/To** | **Conventional** | | | | **ICG** | | | |
| --- | --- | --- | --- | --- | --- | --- | --- | --- |
|  | **Eu** | **T** | **P** | **D** | **Eu** | **T** | **P** | **D** |
| Eu | 0.992077 | 0.000000 | 0.000000 | 0.007923 | 0.992077 | 0.000000 | 0.000000 | 0.007923 |
| T | 0.649061 | 0.000000 | 0.343016 | 0.007923 | 0.959095 | 0.000000 | 0.032982 | 0.007923 |
| P | 0.000000 | 0.000000 | 0.986254 | 0.013746 | 0.000000 | 0.000000 | 0.986254 | 0.013746 |
| D | 0.000000 | 0.000000 | 0.000000 | 1.000000 | 0.000000 | 0.000000 | 0.000000 | 1.000000 |

1. Annual (Years 20 – 24)

| **From/To** | **Conventional** | | | | **ICG** | | | |
| --- | --- | --- | --- | --- | --- | --- | --- | --- |
|  | **Eu** | **T** | **P** | **D** | **Eu** | **T** | **P** | **D** |
| Eu | 0.987277 | 0.000000 | 0.000000 | 0.012723 | 0.987277 | 0.000000 | 0.000000 | 0.012723 |
| T | 0.645920 | 0.000000 | 0.341357 | 0.012723 | 0.954454 | 0.000000 | 0.032823 | 0.012723 |
| P | 0.000000 | 0.000000 | 0.977967 | 0.022033 | 0.000000 | 0.000000 | 0.977967 | 0.022033 |
| D | 0.000000 | 0.000000 | 0.000000 | 1.000000 | 0.000000 | 0.000000 | 0.000000 | 1.000000 |

1. Annual (Years 25 – 29)

| **From/To** | **Conventional** | | | | **ICG** | | | |
| --- | --- | --- | --- | --- | --- | --- | --- | --- |
|  | **Eu** | **T** | **P** | **D** | **Eu** | **T** | **P** | **D** |
| Eu | 0.978296 | 0.000000 | 0.000000 | 0.021704 | 0.978296 | 0.000000 | 0.000000 | 0.021704 |
| T | 0.640044 | 0.000000 | 0.338251 | 0.021704 | 0.945771 | 0.000000 | 0.032524 | 0.021704 |
| P | 0.000000 | 0.000000 | 0.962538 | 0.037462 | 0.000000 | 0.000000 | 0.962538 | 0.037462 |
| D | 0.000000 | 0.000000 | 0.000000 | 1.000000 | 0.000000 | 0.000000 | 0.000000 | 1.000000 |

1. Annual (Years 30 – 35

| **From/To** | **Conventional** | | | | **ICG** | | | |
| --- | --- | --- | --- | --- | --- | --- | --- | --- |
|  | **Eu** | **T** | **P** | **D** | **Eu** | **T** | **P** | **D** |
| Eu | 0.959798 | 0.000000 | 0.000000 | 0.040202 | 0.959798 | 0.000000 | 0.000000 | 0.040202 |
| T | 0.627942 | 0.000000 | 0.331855 | 0.040202 | 0.927888 | 0.000000 | 0.031909 | 0.040202 |
| P | 0.000000 | 0.000000 | 0.931092 | 0.068908 | 0.000000 | 0.000000 | 0.931092 | 0.068908 |
| D | 0.000000 | 0.000000 | 0.000000 | 1.000000 | 0.000000 | 0.000000 | 0.000000 | 1.000000 |

**Estimation of Training Costs**

Although vendor-provided installation and training are usually bundled at no extra financial charge to hospitals, the adoption of new fluorescence imaging technology requires the allocation of staff time for initial familiarization and in-service sessions. From a hospital resource utilization perspective, these represent an opportunity cost that should be reflected in the model.

- **Team composition for a tertiary care hospital:**
- 1 senior endocrine surgeon
- 1 assistant surgeon
- 2 scrub nurses
- 1 anaesthesiologist
- **Training duration (vendor-supported, in-service sessions):**
- Surgeons: 12 hours each
- Scrub nurses: 6 hours each
- Anaesthesiologist: 3 hours
- **Unit cost assumptions and opportunity cost calculation:**

1. Opportunity Costs by Professional Category

| **Role** | **Count** | **Hours** | **Rate (€/h)** | **Subtotal (€)** |
| --- | --- | --- | --- | --- |
| Senior surgeon | 1 | 12 | 24.53 | 294.37 |
| Assistant surgeon | 1 | 12 | 24.53 | 294.37 |
| Scrub nurse (each) | 2 | 6 | 18.49 | 221.83 |
| Anaesthesiologist | 1 | 3 | 24.53 | 73.59 |

- **Total training opportunity costs:**

In Catalonia, high-complexity care is delivered through a limited number of tertiary hospitals integrated within the Catalan Health Service (CatSalut), either directly managed by the *Institut Català de la Salut* (ICS) or by consortia and foundations under the SISCAT network. For the purpose of this analysis, we consider as tertiary hospitals those centres providing a full portfolio of highly specialized services, with teaching and research functions. These include:

1. Hospital Universitari Vall d’Hebron (Barcelona, ICS)
2. Hospital Universitari de Bellvitge (L’Hospitalet de Llobregat, ICS)
3. Hospital Universitari Germans Trias i Pujol (Badalona, ICS)
4. Hospital Clínic de Barcelona (Consorci)
5. Hospital de Sant Pau (Fundación Privada)
6. Hospital del Mar – Parc de Salut Mar (Consorci)
7. Hospital Universitari Doctor Josep Trueta (Girona, ICS)
8. Hospital Universitari Arnau de Vilanova de Lleida (ICS)
9. Hospital Joan XXIII (Tarragona, ICS).

Together, these nine tertiary hospitals constitute the main referral centres for complex endocrine surgery, including total thyroidectomy for thyroid cancer, and thus represent the most relevant setting for the economic evaluation of fluorescence-guided thyroid surgery in Catalonia. That said, Supplemental Table S23 lists the total costs for the healthcare system:

1. Total Opportunity Cost for the Spanish National Health System

| **Metric** | **Total (€)** |
| --- | --- |
| Per-hospital one-off (€) | € 884.16 |
| System one-off (€) | € 7,957.38 |
| System annual amortized (€) – in 10 years | € 795.74 |
| Cost per case (€) | € 0.56 |

**Mapped QALYs**

Utilities for model health states were obtained by mapping published SF-36 domain scores to EQ-5D-3L utilities using the Rowen *et al*. (2009) mapping function (UK EQ-5D-3L TTO tariff) ^1^, that is, using the proposed random-effects (*i.e*., the model treats differences between individuals (patients) as **random variation**, allowing generalization beyond the sampled cohort) generalized least squares (GLS) model 3, a regression method similar to ordinary least squares, but that adjusts for correlated errors). Inputs were rescaled to 0–1 before model evaluation, and predictions were clamped to [−0.594, 1.000].

GLS model 3 is the recommended version by Rowen *et al*. because it captures the **nonlinear** relationship between SF-36 and EQ-5D, as it includes linear terms (the direct contribution of each SF-36 domain), squared terms (allow the relationship to curve —*e.g.*, very high/low scores affect utility differently), and interaction terms (capture how two domains jointly influence HRQoL —*e.g*., pain × physical function).

Utilities were mapped from three studies that report SF-36 domain means in HypoPT populations:

- Jørgensen *et al*. (2024) ^2^ — Base case
- Cherchir *et al*. (2023) ^3^ — Sensitivity analysis
- Astor *et al*. (2016) ^4^ — Sensitivity analysis

1. Mean (SD) SF-36 Scores

| **Study** | **Group** | **PCS** | **MCS** | **PF** | **RP** | **BP** | **GH** | **VT** | **SF** | **RE** | **MH** |
| --- | --- | --- | --- | --- | --- | --- | --- | --- | --- | --- | --- |
| Jørgensen e*t al.* (2024) ^2^ * | **HypoPT** | **—** | **—** | 66.7 (5.5) | 48.2 (12.1) | 54.9 (9.0) | 33.2 (3.3) | 31.7 (7.4) | 70.9 (7.2) | 61.9 (12.4) | 63.1 (4.4) |
|  | **Eu** | **—** | **—** | **81.9 (3.7)** | **80.8 (6.0)** | **77.3 (5.0)** | **46.9 (2.4)** | **58.2 (3.6)** | **85.8 (3.6)** | **85.8 (5.9)** | **77.3 (2.8)** |
| Cherchir *et al*. (2023) ^3^ *† | **HypoPT** | **—** | **—** | **75.0 (18.2)** | **34.4 (31.1)** | **47.4 (21.4)** | **25.8 (17.2)** | **29.3 (17.1)** | **40.7 (24.5)** | **22.5 (19.3)** | **41.6 (13.7)** |
|  | **Eu** | **—** | **—** | **88.6 (16.2)** | **67.9 (29.2)** | **71.9 (21.5)** | **48.5 (15.3)** | **52.3 (12.9)** | **70.3 (20.8)** | **66.5 (24.5)** | **62.5 (11.8)** |
| Astor *et al*. (2016) ^4^ *† | **HypoPT** | **—** | **—** | **72.2 (24.4)** | **39.2 (43.1)** | **55.3 (26.0)** | **48.7 (27.1)** | **40.0 (22.6)** | **67.4 (27.4)** | **63.9 (42.8)** | **70.2 (19.0)** |
|  | **Eu** | **—** | **—** | 87.2 (18.7) | 77.9 (35.8) | 75.1 (26.0) | 76.8 (22.0) | 60.0 (20.8) | 85.5 (22.2) | 81.6 (32.4) | 78.8 (16.5) |

QOL, quality of life; PCS, physical component summary; MCS, mental component summary; PF, physical functioning; RP, role

1. Mapped EQ-5D Values

| **Study** | **Group** | **Mapped EQ-5D** |
| --- | --- | --- |
| Jørgensen e*t al.* (2024) ^2^ * | **HypoPT** | 0.603424 |
|  | **Eu** | 0.797211 |
| Cherchir *et al*. (2023) ^3^ *† | **HypoPT** | 0.508243 |
|  | **Eu** | 0.738014 |
| Astor *et al*. (2016) ^4^ *† | **HypoPT** | 0.619081 |
|  | **Eu** | 0.807599 |

**Supplementary Results**

**Means Breakdown by 5-Year Horizons**

1. Five-Year Cumulative Costs

| **Strategy** | **Mean Costs (€)** | **Lower 95% UI** | **Upper 95% UI** |
| --- | --- | --- | --- |
| **Conventional** |  |  |  |
| 5 years | 149,342,530.47 | 126,752,363.39 | 174,017,596.18 |
| 10 years | 256,356,888.87 | 227,044,458.31 | 287,841,966.80 |
| 15 years | 349,804,971.02 | 316,056,305.30 | 385,288,278.87 |
| 20 years | 430,451,325.64 | 393,455,819.78 | 468,723,862.36 |
| 25 years | 500,347,660.94 | 462,268,183.05 | 540,036,456.70 |
| 30 years | 560,913,207.12 | 522,007,405.27 | 601,938,027.49 |
| 35 years | 612,917,052.66 | 571,326,489.41 | 655,235,360.66 |
| **ICG** |  |  |  |
| 5 years | 147,688,602.68 | 125,340,714.03 | 172,073,787.24 |
| 10 years | 249,559,261.70 | 220,909,105.18 | 280,197,985.68 |
| 15 years | 335,702,310.83 | 303,140,159.64 | 369,929,328.18 |
| 20 years | 407,817,677.29 | 372,696,377.52 | 444,507,598.08 |
| 25 years | 468,638,168.14 | 432,719,965.50 | 506,139,716.18 |
| 30 years | 520,091,326.62 | 483,365,474.76 | 558,365,723.00 |
| 35 years | 563,387,590.60 | 524,843,291.01 | 602,969,180.91 |

1. Five-Year Cumulative QALYs (95% UI) – Jørgensen *et al*. (2024) ^2^

| **Strategy** | **Mean QALYs** | **Lower 95% UI** | **Upper 95% UI** |
| --- | --- | --- | --- |
| **Conventional** |  |  |  |
| 5 years | 12,608.51 | 10,277.39 | 15,206.83 |
| 10 years | 44,072.98 | 38,183.09 | 50,409.89 |
| 15 years | 87,764.72 | 78,296.80 | 98,060.38 |
| 20 years | 138,590.98 | 125,626.55 | 152,344.26 |
| 25 years | 192,568.07 | 176,774.72 | 209,406.00 |
| 30 years | 247,031.03 | 228,930.23 | 265,914.63 |
| 35 years | 299,100.07 | 278,751.34 | 320,667.88 |
| **ICG** |  |  |  |
| 5 years | 12,762.17 | 10,387.41 | 15,413.68 |
| 10 years | 45,239.49 | 39,172.34 | 51,772.93 |
| 15 years | 90,540.68 | 80,740.47 | 101,211.40 |
| 20 years | 143,376.09 | 129,954.50 | 157,640.37 |
| 25 years | 199,608.13 | 183,235.45 | 217,107.82 |
| 30 years | 256,475.01 | 237,676.76 | 276,087.12 |
| 35 years | 310,989.22 | 289,815.24 | 333,450.71 |

1. Five-Year Cumulative QALYs (95% UI) for Scenario 1 – Cherchir *et al*. (2023) ^3^

| **Strategy** | **Mean QALYs** | **Lower 95% UI** | **Upper 95% UI** |
| --- | --- | --- | --- |
| **Conventional** |  |  |  |
| 5 years | 11,500.85 | 9,372.85 | 13,872.20 |
| 10 years | 40,256.22 | 34,873.04 | 46,047.05 |
| 15 years | 80,202.63 | 71,548.53 | 89,616.40 |
| 20 years | 126,681.60 | 114,829.37 | 139,260.65 |
| 25 years | 176,049.00 | 161,612.83 | 191,448.85 |
| 30 years | 225,867.28 | 209,322.91 | 243,136.67 |
| 35 years | 273,502.77 | 254,894.21 | 293,231.85 |
| **ICG** |  |  |  |
| 5 years | 11,803.25 | 9,606.44 | 14,256.25 |
| 10 years | 41,854.60 | 36,240.97 | 47,900.14 |
| 15 years | 83,775.85 | 74,706.56 | 93,649.03 |
| 20 years | 132,671.17 | 120,251.72 | 145,871.83 |
| 25 years | 184,711.02 | 169,559.53 | 200,904.83 |
| 30 years | 237,339.18 | 219,944.08 | 255,488.15 |
| 35 years | 287,790.62 | 268,195.82 | 308,577.64 |

1. Five-Year Cumulative QALYs (95% UI) for Scenario 2 – Astor *et al*. (2016) ^4^

| **Strategy** | **Mean QALYs** | **Lower 95% UI** | **Upper 95% UI** |
| --- | --- | --- | --- |
| **Conventional** |  |  |  |
| 5 years | 12,799.34 | 10,433.16 | 15,436.93 |
| 10 years | 44,731.49 | 38,754.57 | 51,162.66 |
| 15 years | 89,070.10 | 79,461.68 | 99,518.08 |
| 20 years | 140,647.37 | 127,490.87 | 154,603.54 |
| 25 years | 195,420.94 | 179,393.24 | 212,507.45 |
| 30 years | 250,686.52 | 232,317.00 | 269,849.47 |
| 35 years | 303,521.84 | 282,872.48 | 325,407.41 |
| **ICG** |  |  |  |
| 5 years | 12,930.22 | 10,524.26 | 15,616.53 |
| 10 years | 45,832.98 | 39,686.31 | 52,452.00 |
| 15 years | 91,727.00 | 81,798.59 | 102,537.58 |
| 20 years | 145,253.52 | 131,656.17 | 159,704.37 |
| 25 years | 202,220.92 | 185,634.05 | 219,949.65 |
| 30 years | 259,831.33 | 240,787.00 | 279,700.08 |
| 35 years | 315,058.23 | 293,607.25 | 337,813.44 |

1. Five-Year ICER Ratio of Means (95% CI) – Jørgensen *et al*. (2024) ^2^

| **Time Horizon** | **ICER (€/QALY gained)** | **Lower 95% CI** | **Upper 95% CI** |
| --- | --- | --- | --- |
| 5 years | –10,763.77 | –10,815.97 | –10,711.91 |
| 10 years | –5,827.34 | –5,841.83 | –5,812.88 |
| 15 years | –5,080.28 | –5,090.13 | –5,070.45 |
| 20 years | –4,730.02 | –4,737.67 | –4,722.38 |
| 25 years | –4,504.15 | –4,510.52 | –4,497.79 |
| 30 years | –4,322.53 | –4,327.94 | –4,317.12 |
| 35 years | –4,165.94 | –4,170.83 | –4,161.05 |

1. Five-Year ICER Ratio of Means (95% CI) for Scenario 1 – Cherchir *et al*. (2023) ^3^

| **Time Horizon** | **ICER (€/QALY gained)** | **Lower 95% CI** | **Upper 95% CI** |
| --- | --- | --- | --- |
| 5 years | –5,469.21 | –5,490.72 | –5,447.80 |
| 10 years | –4,252.82 | –4,263.05 | –4,242.61 |
| 15 years | –3,946.77 | –3,954.29 | –3,939.25 |
| 20 years | –3,778.84 | –3,784.88 | –3,772.81 |
| 25 years | –3,660.75 | –3,665.88 | –3,655.63 |
| 30 years | –3,558.42 | –3,562.84 | –3,554.01 |
| 35 years | –3,466.54 | –3,470.59 | –3,462.50 |

1. Five-Year ICER Ratio of Means (95% CI) for Scenario 2 – Astor *et al*. (2016) ^4^

| **Time Horizon** | **ICER (€/QALY gained)** | **Lower 95% CI** | **Upper 95% CI** |
| --- | --- | --- | --- |
| 5 years | –12,636.92 | –12,702.94 | –12,571.39 |
| 10 years | –6,171.30 | –6,186.77 | –6,155.88 |
| 15 years | –5,307.94 | –5,318.27 | –5,297.63 |
| 20 years | –4,913.79 | –4,921.76 | –4,905.83 |
| 25 years | –4,663.17 | –4,669.78 | –4,656.58 |
| 30 years | –4,463.94 | –4,469.54 | –4,458.35 |
| 35 years | –4,293.33 | –4,298.38 | –4,288.28 |

1. Five-Year NMB (95% UI) – Jørgensen *et al*. (2024) ^2^

| **Strategy** | **NMB (€)** | **Lower 95% UI** | **Upper 95% UI** |
| --- | --- | --- | --- |
| **Conventional** |  |  |  |
| 5 years | 228,912,907.66 | 149,785,182.50 | 315,673,660.37 |
| 10 years | 1,065,832,508.26 | 877,803,401.03 | 1,267,329,982.32 |
| 15 years | 2,283,136,536.84 | 1,989,095,329.23 | 2,602,029,445.48 |
| 20 years | 3,727,278,036.78 | 3,329,199,838.44 | 4,148,554,509.24 |
| 25 years | 5,276,694,498.88 | 4,794,226,660.52 | 5,793,497,269.83 |
| 30 years | 6,850,017,549.70 | 6,297,282,182.75 | 7,424,327,860.50 |
| 35 years | 8,360,085,060.55 | 7,738,913,570.08 | 9,018,378,054.12 |
| **ICG** |  |  |  |
| 5 years | 235,176,542.57 | 155,058,724.12 | 323,096,445.97 |
| 10 years | 1,107,625,328.35 | 915,235,511.81 | 1,314,796,292.62 |
| 15 years | 2,380,518,055.23 | 2,077,706,088.51 | 2,709,076,165.20 |
| 20 years | 3,893,464,990.04 | 3,480,682,144.40 | 4,328,629,301.56 |
| 25 years | 5,519,605,623.88 | 5,021,006,314.01 | 6,054,223,919.92 |
| 30 years | 7,174,158,902.97 | 6,602,457,106.39 | 7,770,012,314.73 |
| 35 years | 8,766,288,967.75 | 8,120,870,286.94 | 9,450,546,272.29 |

1. Five-Year NMB (95% UI) for Scenario 1 – Cherchir *et al*. (2023) ^3^

| **Strategy** | **NMB (€)** | **Lower 95% UI** | **Upper 95% UI** |
| --- | --- | --- | --- |
| **Conventional** |  |  |  |
| 5 years | 195,682,853.60 | 122,392,362.84 | 275,556,077.34 |
| 10 years | 951,329,757.48 | 778,189,480.77 | 1,136,515,397.96 |
| 15 years | 2,056,273,961.99 | 1,785,908,772.14 | 2,349,195,467.58 |
| 20 years | 3,369,996,580.63 | 3,003,943,375.59 | 3,756,543,418.24 |
| 25 years | 4,781,122,340.17 | 4,339,708,115.54 | 5,254,395,485.94 |
| 30 years | 6,215,105,043.86 | 5,707,673,496.26 | 6,740,940,422.38 |
| 35 years | 7,592,166,053.06 | 7,022,783,637.43 | 8,195,144,561.43 |
| **ICG** |  |  |  |
| 5 years | 206,408,986.25 | 131,346,765.73 | 288,440,097.04 |
| 10 years | 1,006,078,855.89 | 826,657,380.40 | 1,198,624,439.10 |
| 15 years | 2,177,573,181.86 | 1,896,007,977.00 | 2,483,171,473.61 |
| 20 years | 3,572,317,495.79 | 3,189,809,685.30 | 3,976,473,698.36 |
| 25 years | 5,072,692,336.37 | 4,611,225,575.84 | 5,568,803,114.15 |
| 30 years | 6,600,084,175.41 | 6,069,102,833.81 | 7,152,625,367.69 |
| 35 years | 8,070,330,948.94 | 7,472,477,086.91 | 8,704,862,674.04 |

1. Five-Year NMB (95% UI) for Scenario 2 – Astor *et al*. (2016) ^4^

| **Strategy** | **NMB (€)** | **Lower 95% UI** | **Upper 95% UI** |
| --- | --- | --- | --- |
| **Conventional** |  |  |  |
| 5 years | 240,218,054.74 | 159,150,052.85 | 329,277,966.76 |
| 10 years | 1,125,430,152.69 | 930,653,952.80 | 1,335,111,897.62 |
| 15 years | 2,416,107,652.17 | 2,109,539,381.78 | 2,748,685,570.07 |
| 20 years | 3,949,787,971.41 | 3,531,948,939.51 | 4,390,744,145.22 |
| 25 years | 5,597,989,350.41 | 5,092,818,894.75 | 6,139,181,063.88 |
| 30 years | 7,274,848,688.13 | 6,695,988,621.59 | 7,878,909,129.64 |
| 35 years | 8,888,359,301.47 | 8,234,681,833.83 | 9,581,401,202.29 |
| **ICG** |  |  |  |
| 5 years | 234,637,708.05 | 154,559,855.35 | 322,466,702.15 |
| 10 years | 1,085,587,820.28 | 895,169,401.95 | 1,289,838,455.12 |
| 15 years | 2,322,297,974.77 | 2,024,149,502.70 | 2,645,764,945.74 |
| 20 years | 3,788,969,846.95 | 3,385,161,182.12 | 4,216,510,948.49 |
| 25 years | 5,362,280,414.53 | 4,872,703,985.98 | 5,886,480,653.91 |
| 30 years | 6,959,682,456.37 | 6,398,668,484.28 | 7,542,737,439.96 |
| 35 years | 8,492,738,210.16 | 7,862,369,097.32 | 9,160,516,393.15 |

**Mean Lifetime Totals Derived from Monte Carlo Simulations**

1. Mean Total Costs (95% UI)

| **Strategy** | **Mean Costs (€)** | **Lower 95% UI** | **Upper 95% UI** |
| --- | --- | --- | --- |
| Conventional | 613,195,075.23 | 572,023,693.83 | 655,951,463.13 |
| ICG | 563,655,998.29 | 525,124,399.67 | 603,534,486.10 |

1. Mean Total QALYs (95% UI) for the Base-Case – Jørgensen *et al*. (2024) ^2^

| **Strategy** | **Mean QALYs** | **Lower 95% UI** | **Upper 95% UI** |
| --- | --- | --- | --- |
| Conventional | 299,107.19 | 278,658.79 | 320,048.37 |
| ICG | 310,996.64 | 289,725.93 | 332,803.15 |

1. Mean Total QALYs (95% UI) for Scenario 1 – Cherchir *et al*. (2023) ^3^

| **Strategy** | **Mean QALYs** | **Lower 95% UI** | **Upper 95% UI** |
| --- | --- | --- | --- |
| Conventional | 273,509.29 | 254,809.97 | 292,662.11 |
| ICG | 287,797.49 | 268,113.25 | 307,978.22 |

1. Mean Total QALYs (95% UI) for Scenario 2 – Astor *et al*. (2016) ^4^

| **Strategy** | **Mean QALYs** | **Lower 95% UI** | **Upper 95% UI** |
| --- | --- | --- | --- |
| Conventional | 303,529.07 | 282,778.51 | 324,779.24 |
| ICG | 315,065.75 | 293,516.77 | 337,157.43 |

1. ICER Ratio of Means (95% CI)

| **Scenario** | **ICER (€/QALY gained)** | **Lower 95% CI** | **Upper 95% CI** |
| --- | --- | --- | --- |
| Base-Case – Jørgensen *et al*. (2024) ^2^ | –4,166.64 | –4,171.49 | –4,161.80 |
| Scenario 1 – Cherchir *et al*. (2023) ^3^ | –3,467.13 | –3,471.14 | –3,463.13 |
| Scenario 2 – Astor *et al*. (2016) ^4^ | –4,294.05 | –4,299.05 | –4,289.06 |

1. NMBs (95% UI)

| **Strategy** | **NMB (€)** | **Lower 95% UI** | **Upper 95% UI** |
| --- | --- | --- | --- |
| **Conventional** |  |  |  |
| Base-Case – Jørgensen *et al*. (2024) ^2^ | 8,360,020,742.78 | 7,736,238,509.92 | 8,998,952,911.97 |
| Scenario 1 – Cherchir *et al*. (2023) ^3^ | 7,592,083,633.50 | 7,020,899,724.66 | 8,178,713,381.56 |
| Scenario 2 – Astor et al. (2016) ^4^ | 8,492,677,022.74 | 7,859,772,095.76 | 9,141,048,600.10 |
| **ICG** |  |  |  |
| Base-Case – Jørgensen *et al*. (2024) ^2^ | 8,766,243,118.39 | 8,118,124,558.84 | 9,430,263,551.06 |
| Scenario 1 – Cherchir *et al*. (2023) ^3^ | 8,070,268,570.93 | 7,470,114,069.65 | 8,684,278,368.10 |
| Scenario 2 – Astor et al. (2016) ^4^ | 8,888,316,352.73 | 8,231,781,595.67 | 9,560,286,800.17 |
| **Incremental NMB** |  |  |  |
| Base-Case – Jørgensen *et al*. (2024) ^2^ | 406,222,375.61 | 381,665,351.59 | 431,454,145.27 |
| Scenario 1 – Cherchir *et al*. (2023) ^3^ | 395,639,330.00 | 371,718,889.83 | 420,149,541.06 |
| Scenario 2 – Astor et al. (2016) ^4^ | 478,184,937.43 | 449,101,873.59 | 508,111,635.14 |

**Deterministic One-Way Sensitivity Analysis**

1. OWSA Summary Results

| **CV** | **Parameter** | **Level** | **ICER** | **Mean INMB** |
| --- | --- | --- | --- | --- |
| 0.1 | Discount rate (annual) | Low | -4,069.03 | 628,995,231.30 |
| 0.1 | Discount rate (annual) | High | -4,275.03 | 271,017,618.06 |
| 0.1 | Annual surgical intake | Low | -4,087.93 | 323,965,102.39 |
| 0.1 | Annual surgical intake | High | -4,218.01 | 488,086,739.35 |
| 0.1 | Share recovering in 0–6 months | Low | -4,198.78 | 454,184,866.31 |
| 0.1 | Share recovering in 0–6 months | High | -4,133.21 | 367,033,234.06 |
| 0.1 | Share recovering in 6–12 months | Low | -4,134.35 | 419,295,316.13 |
| 0.1 | Share recovering in 6–12 months | High | -4,198.09 | 393,420,798.93 |
| 0.1 | Share recovering within 12 months (total) | Low | -4,164.84 | 345,841,593.41 |
| 0.1 | Share recovering within 12 months (total) | High | -4,166.76 | 458,424,979.01 |
| 0.1 | RR permanent HypoPT at 12m (ICG vs Conventional) | Low | -4,405.26 | 181,512,528.71 |
| 0.1 | RR permanent HypoPT at 12m (ICG vs Conventional) | High | -4,676.45 | 96,129,084.80 |
| 0.1 | Hazard ratio for mortality (Permanent vs EuPT) | Low | -4,487.94 | 387,251,576.34 |
| 0.1 | Hazard ratio for mortality (Permanent vs EuPT) | High | -3,880.60 | 424,601,503.44 |
| 0.1 | Utility source (mapped EQ-5D) | Cherchi et al. ^3^ | -3,466.59 | 477,955,362.19 |
| 0.1 | Utility source (mapped EQ-5D) | Astor et al. ^4^ | -4,293.39 | 395,447,743.61 |
| 0.1 | ER severity mix (mild:severe) | 70:30 | -4,190.08 | 406,312,067.66 |
| 0.1 | ER severity mix (mild:severe) | 90:10 | -4,141.92 | 405,739,774.09 |
|  |  |  |  |  |
| 0.2 | Discount rate (annual) | Low | -4,071.20 | 628,535,909.07 |
| 0.2 | Discount rate (annual) | High | -4,277.31 | 270,820,470.97 |
| 0.2 | Annual surgical intake | Low | -4,090.11 | 323,728,611.80 |
| 0.2 | Annual surgical intake | High | -4,220.26 | 487,731,310.44 |
| 0.2 | Share recovering in 0–6 months | Low | -4,201.02 | 453,854,005.95 |
| 0.2 | Share recovering in 0–6 months | High | -4,135.41 | 366,765,532.16 |
| 0.2 | Share recovering in 6–12 months | Low | -4,136.55 | 418,989,502.53 |
| 0.2 | Share recovering in 6–12 months | High | -4,200.33 | 393,134,199.73 |
| 0.2 | Share recovering within 12 months (total) | Low | -4,167.06 | 345,589,497.66 |
| 0.2 | Share recovering within 12 months (total) | High | -4,168.97 | 458,090,829.38 |
| 0.2 | RR permanent HypoPT at 12m (ICG vs Conventional) | Low | -4,407.60 | 181,380,810.40 |
| 0.2 | RR permanent HypoPT at 12m (ICG vs Conventional) | High | -4,678.94 | 96,059,675.41 |
| 0.2 | Hazard ratio for mortality (Permanent vs EuPT) | Low | -4,490.32 | 386,970,989.78 |
| 0.2 | Hazard ratio for mortality (Permanent vs EuPT) | High | -3,882.67 | 424,290,333.24 |
| 0.2 | Utility source (mapped EQ-5D) | Cherchi et al. ^3^ | -3,468.44 | 477,602,306.89 |
| 0.2 | Utility source (mapped EQ-5D) | Astor et al. ^4^ | -4,295.67 | 395,160,180.51 |
| 0.2 | ER severity mix (mild:severe) | 70:30 | -4,192.31 | 406,016,032.96 |
| 0.2 | ER severity mix (mild:severe) | 90:10 | -4,144.12 | 405,443,889.28 |
|  |  |  |  |  |
| 0.3 | Discount rate (annual) | Low | -4,073.25 | 628,085,220.24 |
| 0.3 | Discount rate (annual) | High | -4,279.46 | 270,627,000.96 |
| 0.3 | Annual surgical intake | Low | -4,092.17 | 323,496,563.13 |
| 0.3 | Annual surgical intake | High | -4,222.38 | 487,382,525.00 |
| 0.3 | Share recovering in 0–6 months | Low | -4,203.13 | 453,529,334.31 |
| 0.3 | Share recovering in 0–6 months | High | -4,137.49 | 366,502,849.90 |
| 0.3 | Share recovering in 6–12 months | Low | -4,138.64 | 418,689,422.97 |
| 0.3 | Share recovering in 6–12 months | High | -4,202.44 | 392,852,961.47 |
| 0.3 | Share recovering within 12 months (total) | Low | -4,169.16 | 345,342,123.34 |
| 0.3 | Share recovering within 12 months (total) | High | -4,171.07 | 457,762,937.48 |
| 0.3 | RR permanent HypoPT at 12m (ICG vs Conventional) | Low | -4,409.82 | 181,251,536.83 |
| 0.3 | RR permanent HypoPT at 12m (ICG vs Conventional) | High | -4,681.29 | 95,991,541.18 |
| 0.3 | Hazard ratio for mortality (Permanent vs EuPT) | Low | -4,492.58 | 386,695,594.86 |
| 0.3 | Hazard ratio for mortality (Permanent vs EuPT) | High | -3,884.62 | 423,985,053.09 |
| 0.3 | Utility source (mapped EQ-5D) | Cherchi et al. ^3^ | -3,470.18 | 477,256,038.06 |
| 0.3 | Utility source (mapped EQ-5D) | Astor et al. ^4^ | -4,297.83 | 394,877,977.20 |
| 0.3 | ER severity mix (mild:severe) | 70:30 | -4,194.42 | 405,725,537.37 |
| 0.3 | ER severity mix (mild:severe) | 90:10 | -4,146.21 | 405,153,550.76 |

1. OWSA of ICER assuming a CV = 0.10

| 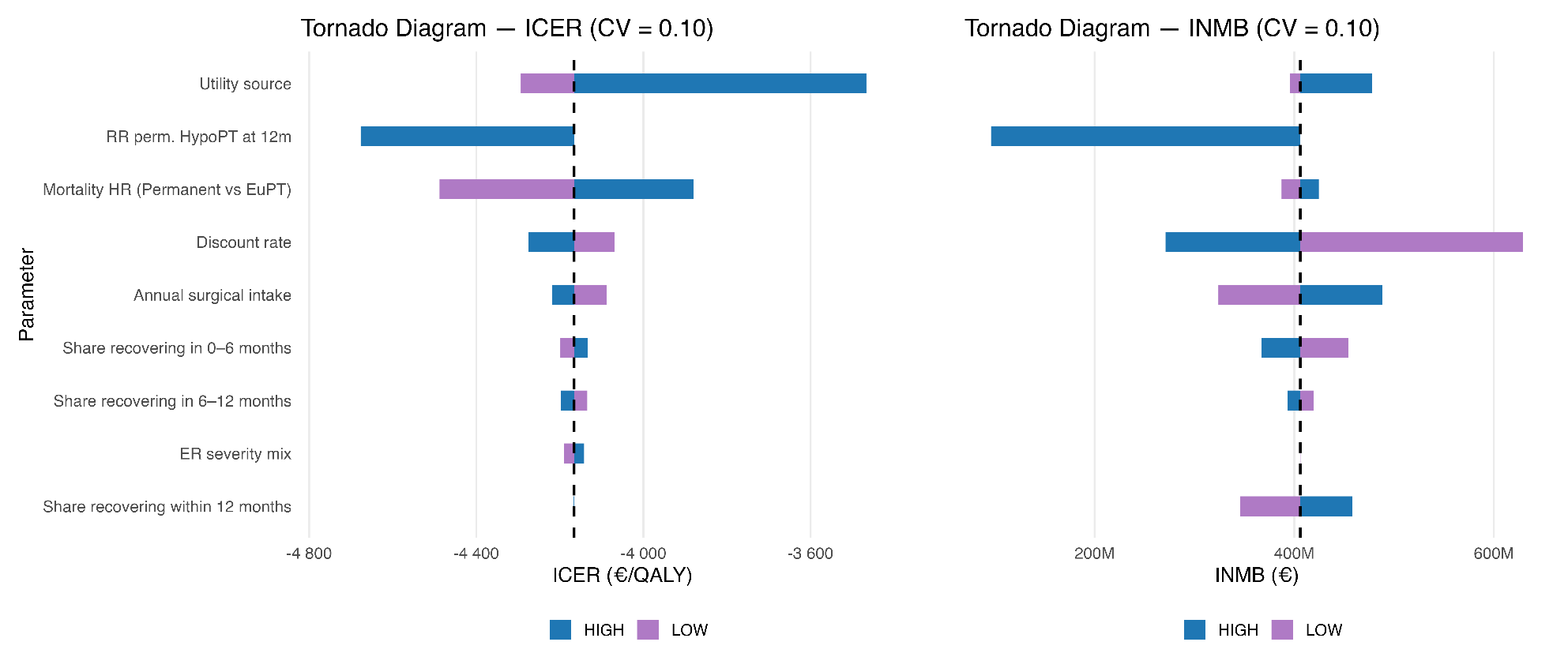 |
| --- |

1. OWSA of ICER assuming a CV = 0.30

| 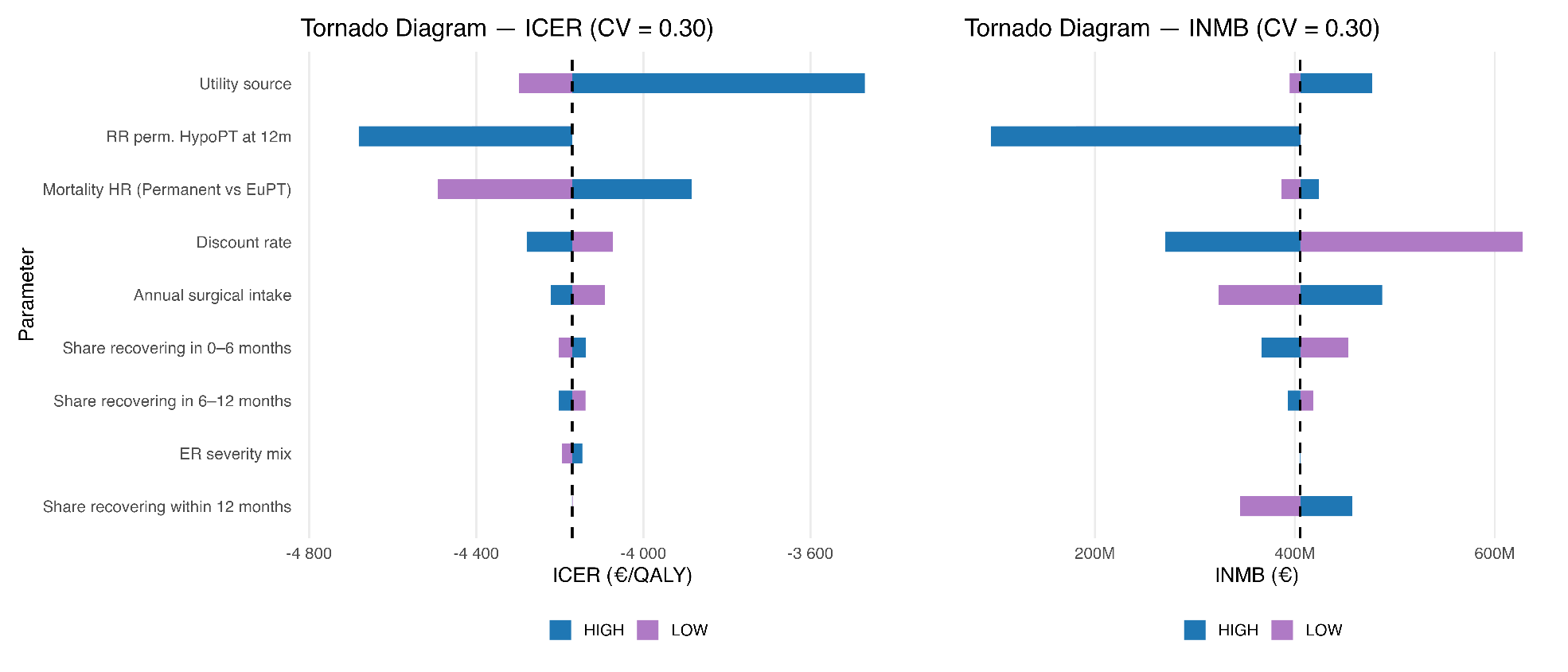 |
| --- |

1. Tornado Diagram assuming a CV = 0.10

|  |
| --- |

1. Tornado Diagram assuming a CV = 0.30

|  |
| --- |

**Bootstrap Validation**

1. Bootstrap Summary Parameters

| **Parameter** | **Mean** | **Lower 95% UI** | **Upper 95% UI** |
| --- | --- | --- | --- |
| Cost Conventional, € | 627,841,984.37 | 624,592,770.09 | 631,058,790.46 |
| Cost ICG, € | 576,371,867.64 | 573,442,161.92 | 579,257,069.87 |
| QALYs Conventional | 309,887.60 | 307,938.51 | 311,838.81 |
| QALYs ICG | 322,273.17 | 320,239.85 | 324,306.30 |
| Delta Cost (ICG - Control), € | -51,470,116.74 | -51,830,911.19 | -51,118,801.37 |
| Delta QALY (ICG - Control) | 12,385.58 | 12,297.81 | 12,472.43 |
| ICER, €/QALY * | -4,155.45 | -4,183.09 | -4,127.99 |
| NMB Conventional, € | 8,668,785,879.57 | 8,610,597,986.14 | 8,726,571,065.58 |
| NMB ICG, € | 9,091,823,355.54 | 9,031,418,291.47 | 9,152,003,325.75 |
| Incremental NMB, € | 423,037,475.97 | 420,190,965.68 | 425,858,828.34 |

1. Mean ∆Cost Frequency

| 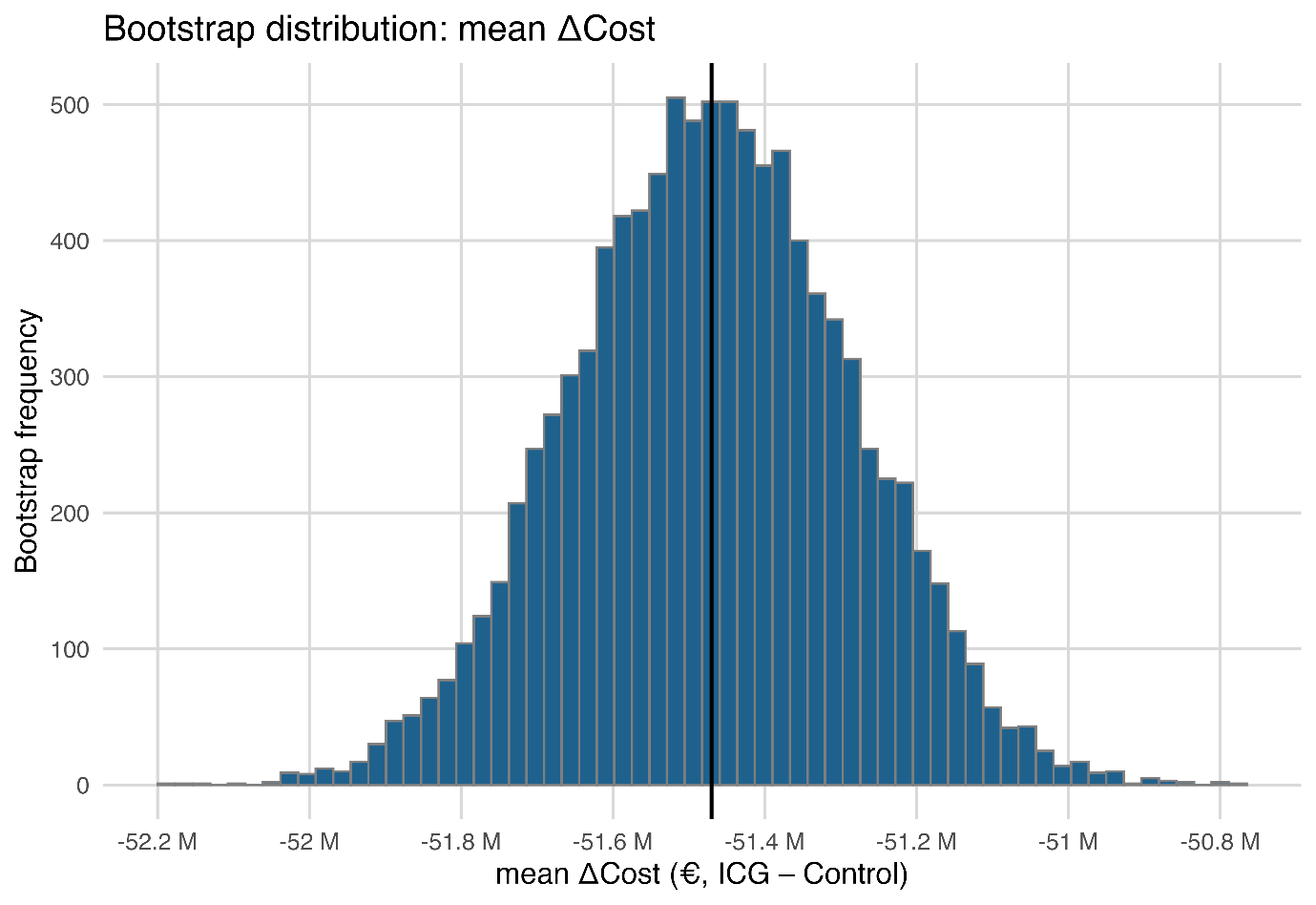 |
| --- |

1. Mean ∆QALY Frequency

| 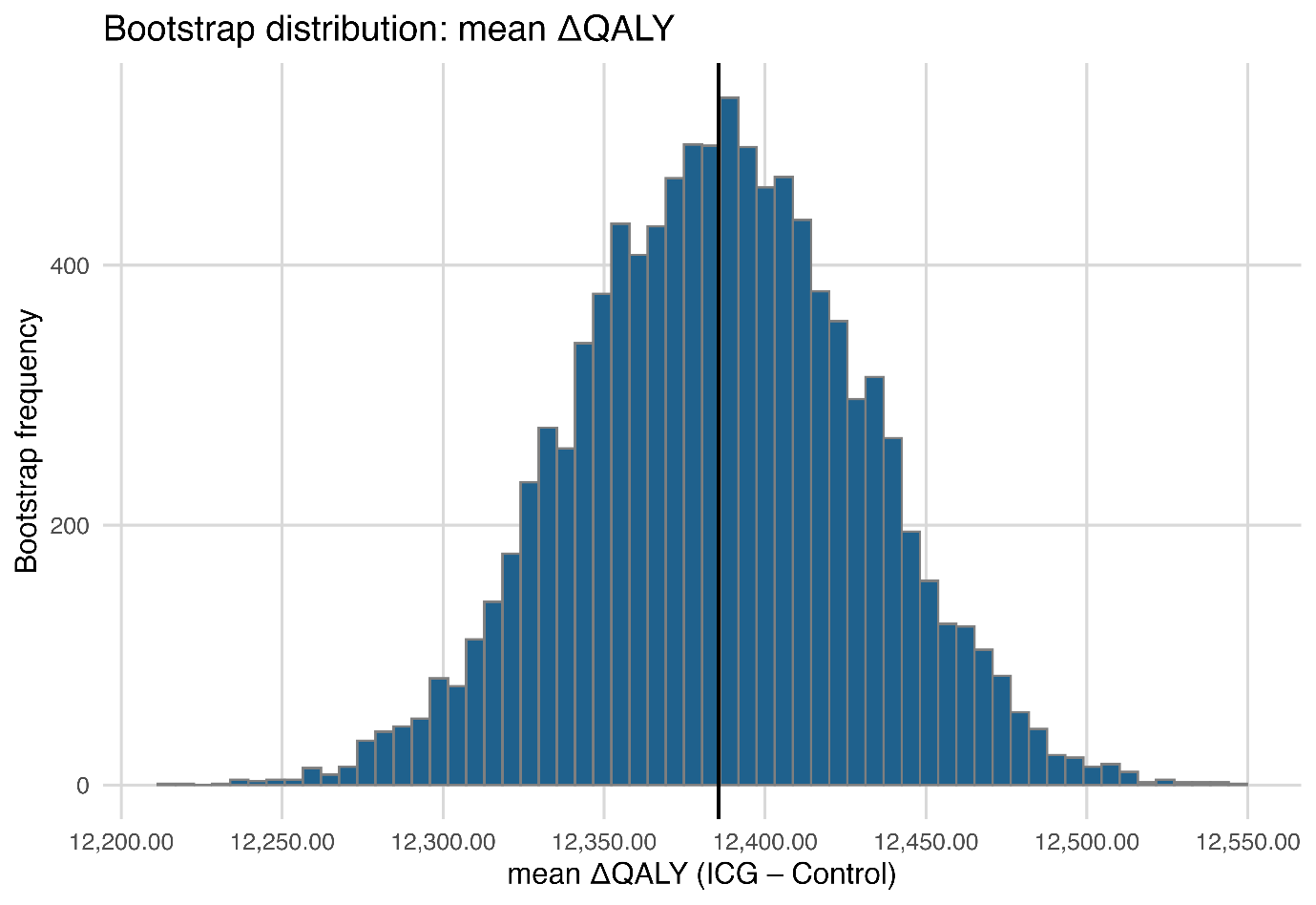 |
| --- |

1. Mean ∆NMB Frequency

| 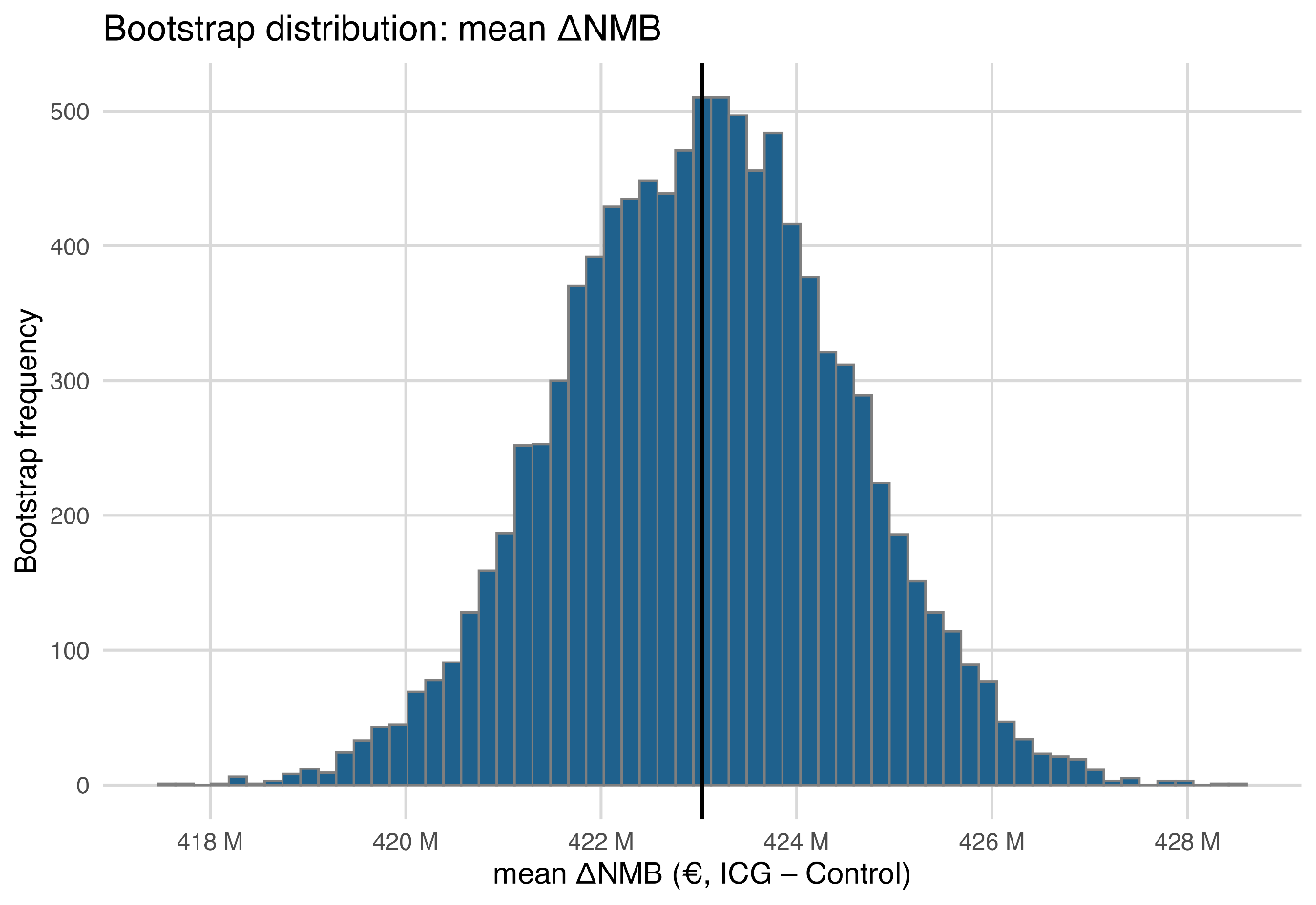 |
| --- |

1. Bootstrap ICER Distribution

| 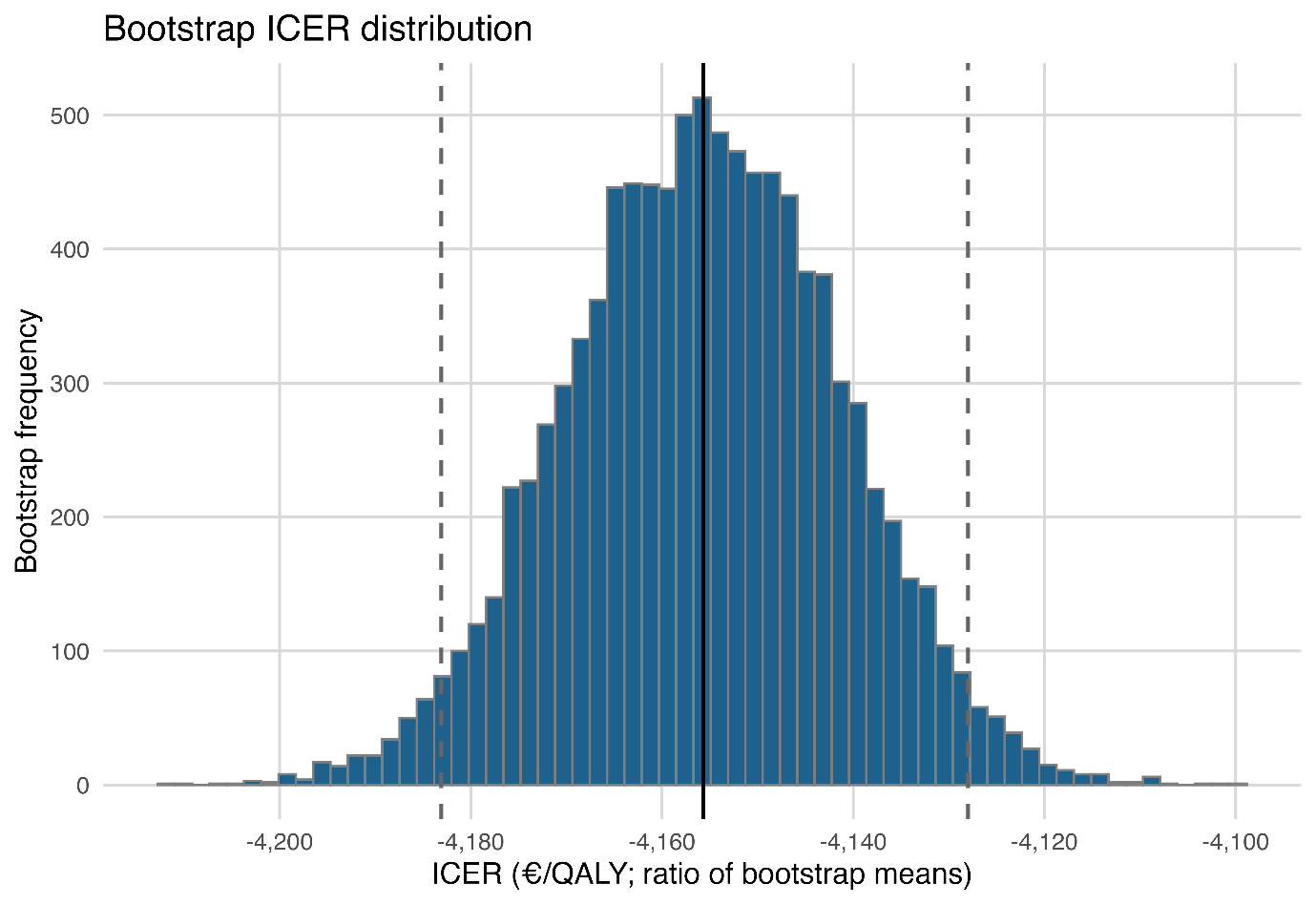 |
| --- |

**References**

1. Rowen D, Brazier J, Roberts J. Mapping SF-36 onto the EQ-5D index: how reliable is the relationship? *Health Qual Life Outcomes* 2009; **7**: 27.

2. Jørgensen CU, Homoe P, Dahl M, Hitz MF. Postoperative Chronic Hypoparathyroidism and Quality of Life After Total Thyroidectomy. *JBMR Plus* 2021; **5**(4): e10479.

3. Cherchir F, Oueslati I, Yazidi M, Chaker F, Chihaoui M. Assessment of quality of life in patients with permanent hypoparathyroidism receiving conventional treatment. *J Diabetes Metab Disord* 2023; **22**(2): 1617-23.

4. Astor MC, Lovas K, Debowska A, et al. Epidemiology and Health-Related Quality of Life in Hypoparathyroidism in Norway. *J Clin Endocrinol Metab* 2016; **101**(8): 3045-53.
